# Multilayered Organization of Jasmonate Signalling in the Regulation of Root Growth

**DOI:** 10.1371/journal.pgen.1005300

**Published:** 2015-06-12

**Authors:** Debora Gasperini, Aurore Chételat, Ivan F. Acosta, Jonas Goossens, Laurens Pauwels, Alain Goossens, René Dreos, Esteban Alfonso, Edward E. Farmer

**Affiliations:** 1 Department of Plant Molecular Biology, University of Lausanne, Lausanne, Switzerland; 2 Department of Plant Systems Biology, Flanders Institute for Biotechnology (VIB), Gent, Belgium; 3 Department of Plant Biotechnology and Bioinformatics, Ghent University, Gent, Belgium; 4 Swiss Institute of Bioinformatics, Lausanne, Switzerland; National University of Singapore and Temasek Life Sciences Laboratory, SINGAPORE

## Abstract

Physical damage can strongly affect plant growth, reducing the biomass of developing organs situated at a distance from wounds. These effects, previously studied in leaves, require the activation of jasmonate (JA) signalling. Using a novel assay involving repetitive cotyledon wounding in *Arabidopsis* seedlings, we uncovered a function of JA in suppressing cell division and elongation in roots. Regulatory JA signalling components were then manipulated to delineate their relative impacts on root growth. The new transcription factor mutant *myc2-322B* was isolated. In vitro transcription assays and whole-plant approaches revealed that *myc2-322B* is a dosage-dependent gain-of-function mutant that can amplify JA growth responses. Moreover, *myc2-322B* displayed extreme hypersensitivity to JA that totally suppressed root elongation. The mutation weakly reduced root growth in undamaged plants but, when the upstream negative regulator NINJA was genetically removed, *myc2-322B* powerfully repressed root growth through its effects on cell division and cell elongation. Furthermore, in a JA-deficient mutant background, *ninja1 myc2-322B* still repressed root elongation, indicating that it is possible to generate JA-responses in the absence of JA. We show that NINJA forms a broadly expressed regulatory layer that is required to inhibit JA signalling in the apex of roots grown under basal conditions. By contrast, MYC2, MYC3 and MYC4 displayed cell layer-specific localisations and MYC3 and MYC4 were expressed in mutually exclusive regions. In nature, growing roots are likely subjected to constant mechanical stress during soil penetration that could lead to JA production and subsequent detrimental effects on growth. Our data reveal how distinct negative regulatory layers, including both NINJA-dependent and -independent mechanisms, restrain JA responses to allow normal root growth. Mechanistic insights from this work underline the importance of mapping JA signalling components to specific cell types in order to understand and potentially engineer the growth reduction that follows physical damage.

## Author Summary

The study of plant development is generally carried out in the absence of physical injury. However, damage to plant organs through biotic and abiotic insult is common in nature. Under these conditions the jasmonate pathway that has a low activity in unstressed vegetative tissues imposes its activity on cell division and elongation. Such jasmonate-dependent growth restriction can strongly impact plant productivity. Taking roots as a model, we show that it is possible to manipulate regulatory layers in jasmonate signalling such that cell division and cell elongation can be constrained differently. This approach may lead to future strategies to alter organ growth. Moreover, during this study we identified a novel mutant in a key regulator of the jasmonate pathway. This mutant generated a positive regulator of jasmonate signalling that was so active that we were able to show that hormone synthesis can be completely uncoupled from hormone responses, suggesting ways to modify traits of potential agronomic importance.

## Introduction

The development, architecture and mass of nascent plant organs are plastic and can be strongly influenced by injury to pre-existing tissues. Wounding reduces plant biomass and damage to young tissues can strongly reduce growth rates, e.g. [1]. In the case of above ground tissues, most of the growth restriction that occurs subsequently to physical damage depends on the activation of the jasmonate (JA) pathway [2–4], which has a pivotal role in controlling herbivore-inducible gene expression and coordinating resource allocation between defence and growth [5, 6]. In contrast to the observation of JA-mediated growth restriction in leaves, root growth responses following damage to aerial organs are so far, not clearly understood. Additionally, there is relatively little knowledge of the cellular organization of JA signalling components in roots. What has emerged to date, however, is that the same basic JA signalling components operate in shoots and roots, although the genetic architecture of the JA pathway appears to be simpler in roots [7].

JA signalling, whether for defence or organ growth restriction, requires the production and perception of low molecular mass lipidic regulators of the JA family, including the biologically active form jasmonoyl-L-isoleucine (JA-Ile) [8, 9]. The transcriptional changes resulting from JA-Ile perception enable plants to modulate the allocation of resources in defense at the expense of growth [3]. In the absence of JA-Ile, JASMONATE ZIM-DOMAIN (JAZ) proteins bind and repress JA-dependent transcription factors (TFs) by recruiting the general co-repressors TOPLESS (TPL) and TPL-Related (TPR) proteins through an interaction with the adaptor protein Novel Interactor of JAZ (NINJA) [10], or directly as in the case of JAZ8 [11]. TPL in turn recruits histone deacetylases and histone methyltransferases to inhibit transcription [12]. Upon stimulation, JA-Ile accumulates and promotes the binding of JAZ proteins to the F-box protein CORONATINE INSENSITIVE 1 (COI1) [13, 14]. This interaction mediates the ubiquitylation and degradation of JAZs [13, 14], liberating TFs to interact with the MED25 subunit of the Mediator complex and recruit RNA polymerase II to JA-responsive genes [15, 16].

**Fig 1:**
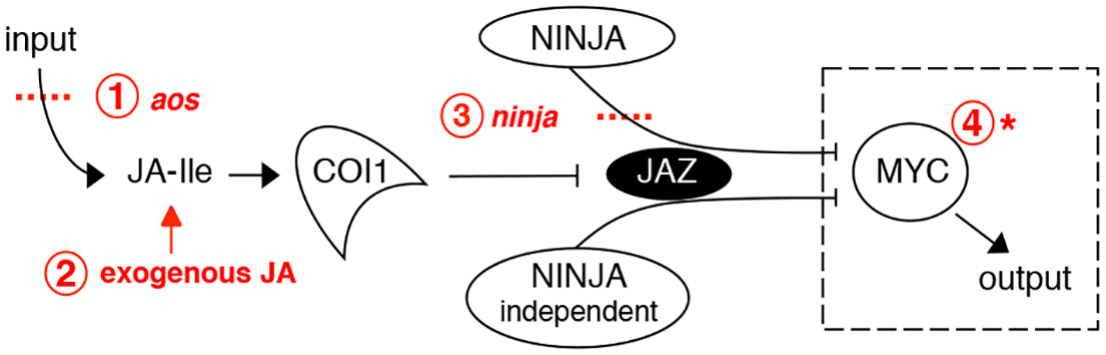
Interventions used in this study to manipulate the jasmonate pathway. Shown in red are: 1. loss-of-function mutation of the JA synthesis gene *allene oxide synthase* (*aos*); 2. treatment with exogenous JA; 3. loss-of-function mutations in the negative regulator *NINJA*; 4. gain-of-function mutation of MYC transcription factors. The dashed box surrounding MYC indicates that it is conceptually possible to use an overactive MYC to drive JA responses in the absence of JA synthesis (step 1) and of negative regulators like NINJA (steps 1 and 3 combined). This was achieved using a novel *myc2* mutant that amplifies JA responses.

Currently, the basic helix-loop-helix (bHLH) MYC2 TF is considered a master regulator of most JA responses and a convergence node with other signalling pathways [17]. It can act as both activator and repressor to regulate diverse aspects of JA-mediated gene expression [18]. Two MYC2-like bHLH proteins (MYC3 and MYC4) also interact with JAZs and act additively with MYC2 in mediating a subset of JA-regulated responses [19, 20]. A simplified scheme for JA signalling is shown in Fig 1. All components shown in the figure can be manipulated to affect the output (defence/growth) of the pathway and, in addition, jasmonate responses can be powerfully activated with exogenous JA. Whatever the upstream manipulation of JA levels or pathway components, much of their activity converges on MYCs. This leads to the theoretical possibility, shown in the dashed box in Fig 1, that strong gain-of-function mutants of MYC2 might be able to recapitulate jasmonate responses in the absence of JA itself. Furthermore, such effects should be facilitated if negative regulatory layers (like that imposed by NINJA) could be removed. Herein, using *Arabidopsis* seedlings we investigated this possibility in the context of the regulatory organisation of JA signalling components and their contribution to root growth.

Although JA-induced root growth inhibition assays have been widely employed to identify JA mutants in *Arabidopsis*, reviewed in [6, 21], the basal root length of those mutants does not differ from wild-type (WT) [22–24]. To date, mutants in *NINJA* represent the sole example of a JA signalling component known to affect normal root growth [7]. However, unlike plants grown in medium supplied with exogenous JA where root growth is inhibited as a consequence of reduced meristem cell number and cell elongation [25], *ninja* mutants display de-repressed JA signalling and shorter roots in the absence of JA due to a defect in cell elongation only [7]. Thus, *ninja* mutants suggest the existence of additional organ- and cell-specific mechanisms of negative regulation that restrict JA signalling responses in the root. We first characterized the effects of endogenous JA on root growth. NINJA, MYC2, MYC3, and MYC4 distribution maps for the root tip were then established, uncovering their overlapping and unique expression domains. A novel gain-of-function allele of *MYC2*, either alone or in combination with *ninja* and JA biosynthesis mutants, revealed the existence of several layers of negative regulation that keep JA responses at bay to allow normal root growth. These results provide a basis for the future engineering of damage- or JA-controlled organ growth, an area of potential importance in agriculture.

## Results

### A JA-dependent shoot-to-root wound signal reduces primary root growth

**Fig 2:**
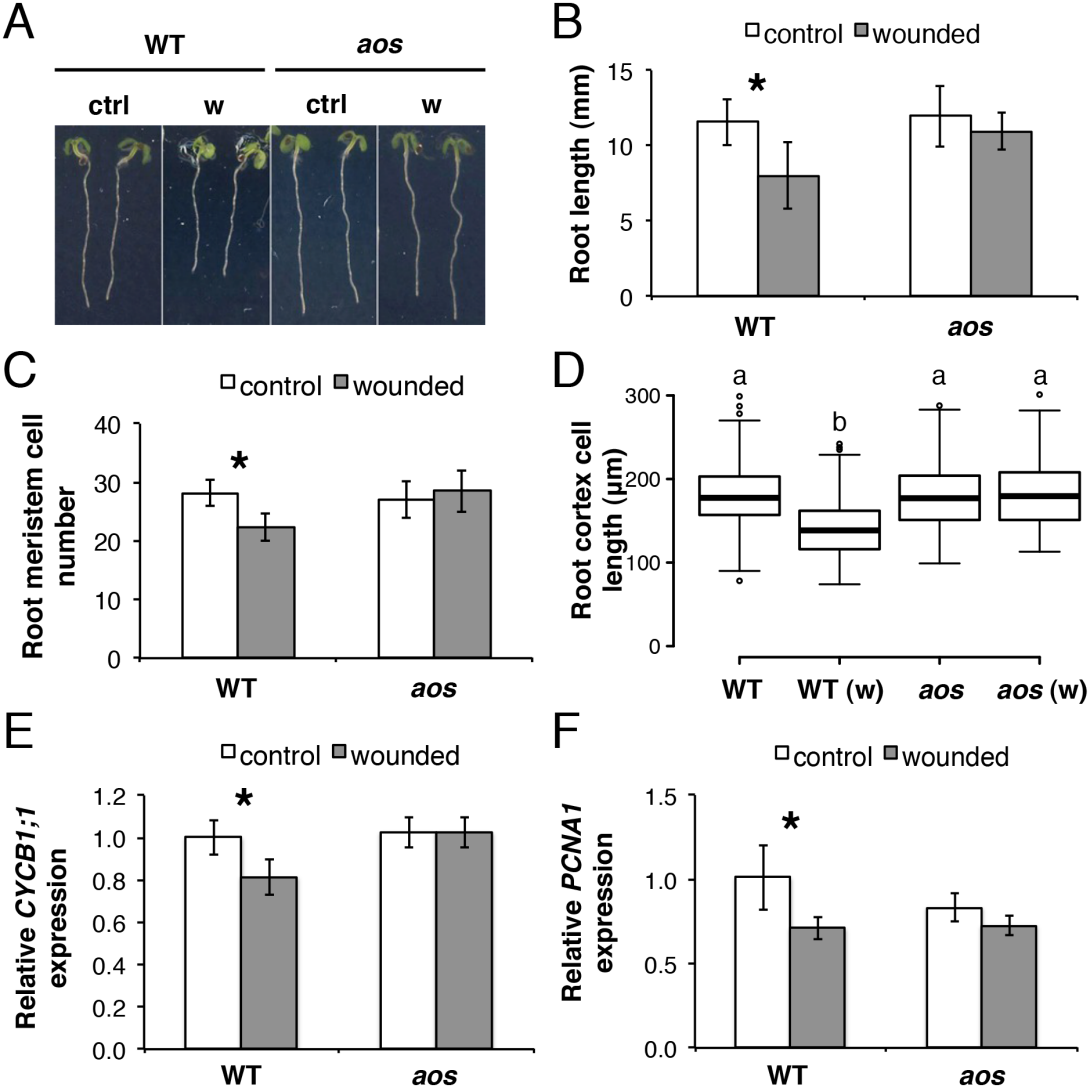
Root growth reduction after repeated shoot wounding is JA dependent. (A-D) Root measurements of 5-day-old (do) WT and *aos* seedlings grown in control conditions or subjected to repetitive cotyledon wounding: (A) representative control (ctrl) and wounded (w) seedlings; (B) primary root length; (C) cortex cell number in the primary root meristem; (D) box plot of cortex cell length in the differentiation zone of the primary root in control and wounded (w) samples of the indicated genotype. Data shown are means (± SD) from 27–55 (B) or 10 plants (C-D). Asterisks: Student’s t test significance compared with untreated controls (*P < 0.001) and letters in (D) indicate statistically significant differences between pairs as determined by Tukey’s HSD test (P < 0.001). (E-F) qRT-PCR of cell cycle marker genes (E) *CYCB1;1* and (F) *PCNA1* in 5-do roots of WT and *aos* seedlings grown in control conditions or subjected to repetitive cotyledon wounding. Root samples were collected 1 h after the 5^th^ cotyledon wound. Transcript levels were normalized to *UBC21* and displayed relative to the expression of the WT control. Bars represent means of three biological replicates (±SD), each containing a pool of roots from ~60 individuals. Asterisks: Student’s t test significance compared with untreated controls (*P < 0.01).

We investigated root growth responses at the seedling stage where mechanical wounding of shoots is known to trigger JA signalling in belowground tissues [7, 26]. Repeated wounding of cotyledons in WT seedlings caused the reduction of root length by decreasing meristem cell number and cell elongation in the differentiation zone (Fig 2A–2D and S1 Fig). Furthermore, the treatment significantly decreased root expression of two cell cycle genes *CYCB1;1* [27] and *PCNA1* [28] (Fig 2E and 2F), confirming the repressive effect of JA signalling on cell division activity in the root meristem. In contrast to the WT, repeated cotyledon wounding did not cause significant morphological or expression changes in roots of the JA-deficient mutant *allene oxide synthase* (*aos*) (Fig 2), indicating that the root growth reduction is JA-dependent. Consistent with an increase in bioactive jasmonates, repeated cotyledon wounding also caused a strong reduction in the levels of the JA-biosensor *Jas9-VENUS* [26] along the root including the meristem (S2 Fig). Thus, a JA-dependent shoot-to-root signal(s) was able to reach the root meristem, activate JA signalling and reduce primary root growth.

### Organization of JA signalling components in the primary root

As there was no information on the spatial organization of JA signalling in the root meristem, which could potentially mediate root growth responses after cotyledon wounding, we investigated the expression domain of NINJA, the only JA-signalling component affecting root growth in undamaged plants [7], and MYC TFs (MYC2, MYC3 and MYC4) known to have a role in the inhibition of root elongation when seedlings are grown in the presence of exogenous JA [19].

*NINJA*_*pro*_ *-GUSPlus* expression was detected in all organs of 5-day-old (do) seedlings: in vascular tissues of cotyledons and hypocotyl, in emerging true leaves, and in the primary root (S3 Fig). The strongest expression was observed in the root particularly in the apical meristem encompassing all cell layers examined. Confirming previous work [25], *MYC2*_*pro*_ *-GUSPlus* was expressed predominantly in the root extending midway into the hypocotyl, and was almost absent in cotyledons. In the division zone of the primary root *MYC2*_*pro*_ *-GUSPlus* activity was found in cells of the epidermis and lateral root cap but not in stele initials, whereas in cells of the elongation and differentiation zones staining was visible in the stele with only a very weak signal from ground tissues (S3 Fig). *MYC3*_*pro*_ *-GUSPlus* and *MYC4*_*pro*_ *-GUSPlus* expression patterns had both distinct and overlapping expression domains to that of *MYC2*_*pro*_ *-GUSPlus* and with respect to each other (S3 Fig). Consistently with [19], *MYC3*_*pro*_ *-GUSPlus* expression was found along the root excluding the division zone with weaker expression in aerial tissues, while the *MYC4* promoter was active in the developed vasculature of the root and it extended to other outer cell layers in the hypocotyl and cotyledons. In addition, we observed *MYC4*_*pro*_ *-GUSPlus* expression in outer layers of the columella and lateral root cap, and a strong *MYC3*_*pro*_ *-GUSPlus* expression in the outer margins of cotyledons, where no apparent *MYC4*_*pro*_ *-GUSPlus* activity was detected.

**Fig 3:**
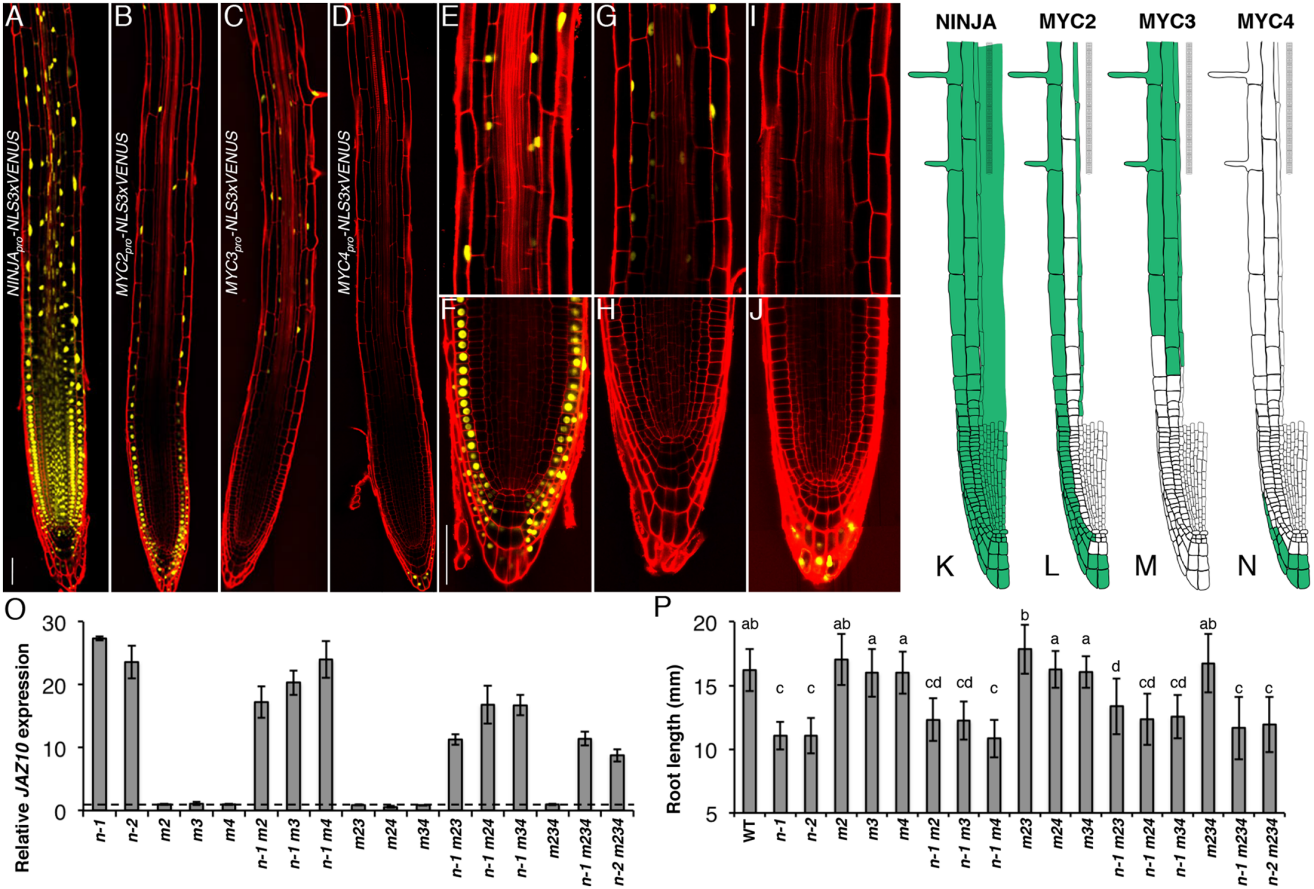
Spatial localization of NINJA, MYC2, MYC3 and MYC4 in the primary root meristem and contribution of the three TFs to the *ninja* mutant phenotype. Expression pattern overviews of (A) *NINJA*_*pro*_ *-NLS3xVENUS*, (B) *MYC2*_*pro*_ *-NLS3xVENUS*, (C) *MYC3*_*pro*_ *-NLS3xVENUS* and (D) *MYC4*_*pro*_ *-NLS3xVENUS* fluorescent reporters in 5-do WT primary roots. Close-ups of *MYC2*_*pro*_ *-NLS3xVENUS* (E and F), *MYC3*_*pro*_ *-NLS3xVENUS* (G and H) and *MYC4*_*pro*_ *-NLS3xVENUS* (I and J) expression in, respectively, the elongation and division zones of the primary root. Confocal microscopy images (A-J) represent merged overlays of the fluorescent (yellow) and propidium iodide (red) stained roots. Scale bars = 50 μm. (K-N) Distribution maps of NINJA, MYC2, MYC3 and MYC4 expression patterns (green) in the primary root meristem based on promoter and protein fusion reporters (S4 Fig). (O) qRT-PCR of basal *JAZ10* expression in 5-do roots of *ninja-1* (*n-1*), *ninja-2* (*n-2*), *myc2* (*m2*), *myc3* (*m3*), *myc4* (*m4*), *ninja-1 myc2* (*n-1 m2*), *ninja-1 myc3* (*n-1 m3*), *ninja-1 myc4* (*n-1 m4*), *myc2 myc3* (*m23*), *myc2 myc4* (*m24*), *myc3 myc4* (*m34*), *ninja-1 myc2 myc3* (*n-1 m23*), *ninja-1 myc2 myc4* (*n-1 m24*), *ninja-1 myc3 myc4* (*n-1 m34*), *myc2 myc3 myc4* (*m234*), *ninja-1 myc2 myc3 myc4* (*n-1 m234*), and *ninja-2 myc2 myc3 myc4* (*n-2 m234*). *JAZ10* transcript levels were normalized to those of *UBC21* and displayed relative to the expression of WT controls that are set to 1 and indicated with a dashed line. Bars represent the means of three biological replicates (±SD), each containing a pool of ~60 roots. Complete qRT-PCR data are in S1 File. (P) Root length of 7-do seedlings of the same genotype as indicated in (O). Data shown are means (± SD) from 22–48 plants; letters above bars indicate statistically significant differences between samples as determined by Tukey’s HSD test (P < 0.01).

Promoter activities of *NINJA* and the three TFs were further characterized at the cellular level in the primary root tip with a nuclear localized fluorescent VENUS reporter protein (Fig 3A–3J). *NINJA*_*pro*_ *-NLS3xVENUS* was strongly expressed in all cells of the primary root apex (Fig 3A). *MYC2*_*pro*_ *-NLS3xVENUS* was expressed in elongating endodermal and epidermal cells of the elongation zone (Fig 3E) as well as epidermal, lateral root cap and columella cells of the root division zone (Fig 3F). A weaker *MYC3*_*pro*_ *-NLS3xVENUS* signal was present in endodermal, cortex and epidermal cells of the elongation and differentiation zones, while it was not detected in cells of the division zone (Fig 3H). *MYC4*_*pro*_ *-NLS3xVENUS* was absent from the elongation and early differentiation zone of the root (Fig 3I), and its expression was restricted to outer layers of the columella and lateral root cap (Fig 3J). The same expression patterns, although with much weaker fluorescent signals for the three MYC TFs, were observed with functional protein fusion reporters driven by endogenous promoters (S4 and S5 Figs). The localization of *NINJA*_*pro*_ *-NLS3xVENUS*, *MYC2*_*pro*_ *-NLS3xVENUS*, *MYC3*_*pro*_ *-NLS3xVENUS* and *MYC4*_*pro*_ *-NLS3xVENUS* florescent reporters did not differ from WT when expressed in the *aos* background (S6 Fig), indicating that the basal expression of *NINJA* and the three TFs in the primary root tip is JA-independent.

A summary of the data is shown in basal distribution maps in the primary root apex (Fig 3K–3N). NINJA was present in all cells of the root tip. MYC2 and MYC3 were coincident in elongating and elongated epidermal and endodermal cells of the elongation and maturation zones of the root meristem. MYC3 was found in cortex cells from the elongation into the maturation zone, while MYC2 was detectable in all epidermal cells in different root zones from the initials on. The expression of MYC2 and MYC4 partially overlapped in the outer layers of the columella and lateral root cap, with MYC2 encompassing also inner cell layers of the two tissues. We did not observe overlapping expression domains for MYC3 and MYC4 in the root tip.

### Contribution of MYC2, MYC3, and MYC4 to the *ninja* root phenotype

Even though MYC2 has a dominant role in root responses to exogenous JA [19, 20, 23], the *ninja* phenotype was not suppressed in a *ninja myc2* double mutant, indicating that other or additional TF(s) are basally repressed by the NINJA-dependent mechanism [7]. Those signalling events are downstream of the COI1 receptor and JA-Ile perception as the *ninja* short root phenotype was still present in a *ninja-1 coi1-1* double mutant background (S7 Fig). We generated multiple *ninja myc* mutant combinations and evaluated their root phenotypes in terms of the early JA signalling marker *JAZ10* [2, 7] and organ length. The 25 times higher-than-WT *JAZ10* expression found in *ninja* roots was only mildly reduced in *ninja-1 myc2* and *ninja-1 myc3* double mutant combinations, while it remained similar to *ninja* in a *ninja-1 myc4* background (Fig 3O). We have previously shown that the *jin1-7* allele of *MYC2* did not suppress ectopic *JAZ10* expression in *ninja* [7], which could be attributed to the different allele used (*jin1-7 vs jin1-2*). On the other hand, *JAZ10* levels were almost halved in a *ninja-1 myc2 myc3* triple mutant but they were not further reduced in *ninja myc2 myc3 myc4* quadruple mutants (Fig 3O). Therefore, the combined activity of MYC2 and MYC3 contributes to approximately 50% of basal *JAZ10* expression in *ninja* roots, and the activity of MYC4 has a minor, if any, role. We also monitored *JAZ10* expression in roots 1 h after wounding one cotyledon (S8 Fig). The higher-than-WT *JAZ10* accumulation of *ninja* mutants was abolished in double and triple mutant combinations with *myc2* (*ninja-1 myc2*, *ninja-1 myc2 myc3*, *ninja-1 myc2 myc4*), while it was persistently higher in double and triple mutants with a WT copy of *MYC2* (*ninja-1 myc3*, *ninja-1 myc4*, *ninja-1 myc3 myc4*).

The roots of *ninja* mutants are 30% shorter than WT [7] (S7 Fig) and we found that this was maintained in *myc2*/ *myc3*/ *myc4* double, triple and quadruple mutant combinations with *ninja* relative to the controls with functional NINJA (Fig 3P). Remaining JA signalling in *ninja myc2 myc3* mutants seems sufficient to repress root growth. Alternatively, it is possible that de-repressed JA signalling is not the direct cause of root growth reduction in *ninja*.

### *ninja* roots display a JA-response transcriptome

To characterize transcriptional changes occurring in the root in the absence of a NINJA-dependent repression mechanism, we conducted a microarray analysis of *ninja-1* versus WT roots. Consistent with a major role of NINJA in repressing JA signalling, many of the 113 genes up-regulated in *ninja-1* roots encoded typical JA-responsive transcripts and several genes involved in secondary metabolism (notably genes encoding the synthesis of triterpenes such as thalianol) (S1 Table). Only 12 genes were down-regulated (including *NINJA*; S1 Table). We identified a larger number of differentially expressed genes in roots than found in a previous study that used adult rosettes of a *NINJA* knock-down line [10], with only 7 genes overlapping between the two datasets (S1 Table). The over-expression of JA response genes in *ninja* could be the result of de-repressed TF(s) normally inhibited by the NINJA-dependent repression mechanism. In an attempt to identify such TF(s) we generated double mutants between *ninja-1* and *WRKY38* and *bHLH025* T-DNA insertion lines, two TFs found over-expressed in the *ninja* root transcriptome (S1 Table). However, *ninja* mutant phenotypes were not suppressed in those double mutant backgrounds (S9 Fig).

### *myc2-322B*: A novel *MYC2* allele

**Fig 4:**
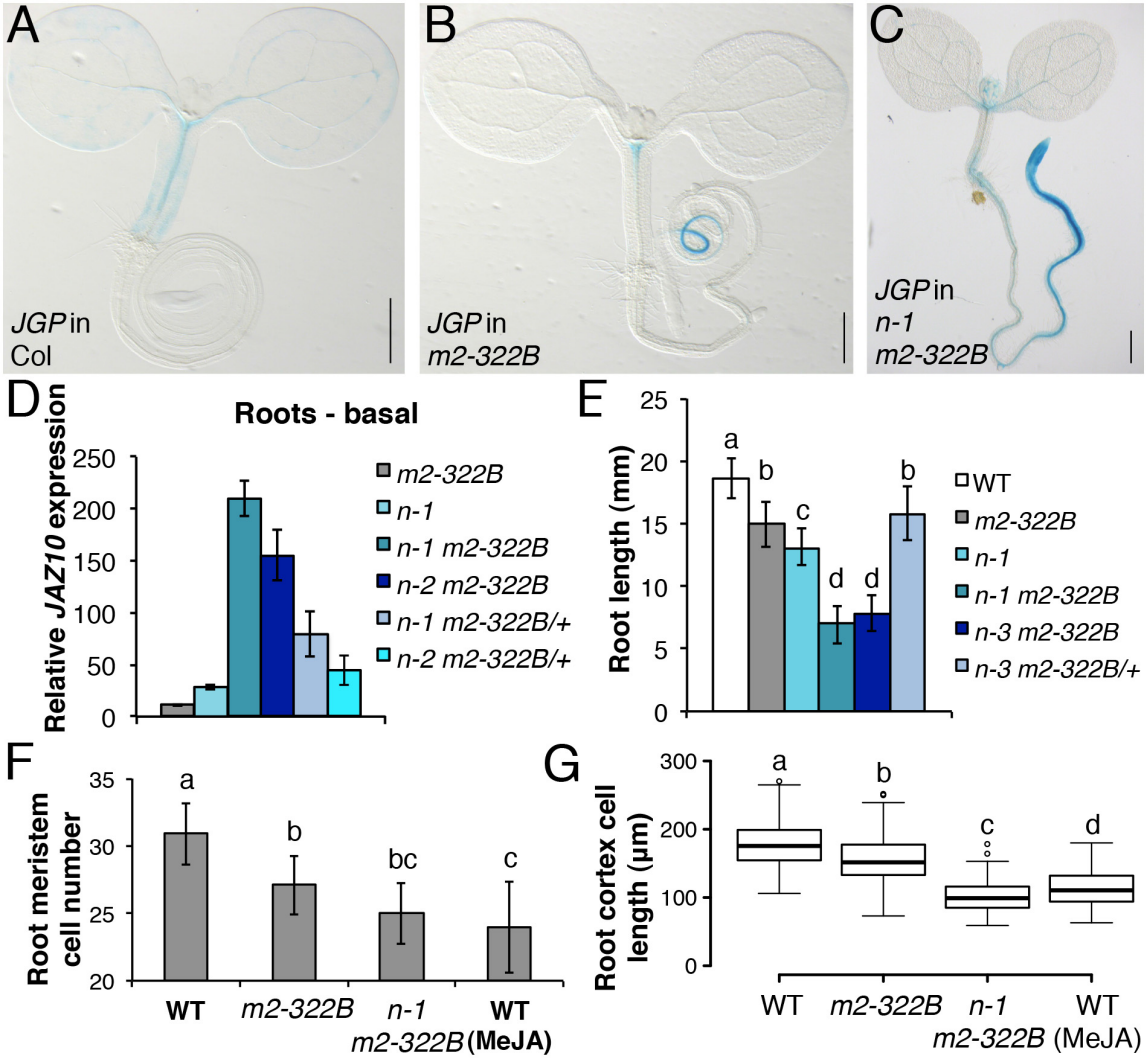
*myc2-322B* exhibits enhanced root JA responses in a NINJA-dependent manner. *JGP* expression in 5-do seedlings of WT (A), *myc2-322B* (B, *m2-322B*) and *ninja-1 myc2-322B* (C, *n-1 m2-322B*). Note the constitutive reporter activity stained in blue (Scale bars = 0.5 mm). (D) qRT-PCR of basal *JAZ10* expression in 5-do roots of *myc2-322B*, *ninja-1* (*n-1*), *ninja-1 myc2-322B* (*n-1 m2-322B*), *ninja-2 myc2-322B* (*n-2 m2-322B*), *ninja-1 myc2-322B/+* (*n-1 m2-322B/+*) and *ninja-2 myc2-322B/+* (*n-2 m2-322B/+*). *JAZ10* transcript levels were normalized to those of *UBC21* and displayed relative to the expression of unwounded WT samples, which are set to 1. Bars represent the means of three biological replicates (±SD), each containing a pool of ~60 roots. Complete qRT-PCR data are in S1 File. (E) Root length quantification of WT and 7-do mutant lines. Data are the means (±SD) from 20–48 plants. Primary root meristem cell number (F) and box plot summary of cortex-cell length (G) in 5-do seedlings of WT and mutant lines grown in control conditions, and of WT grown in presence of 25 μM MeJA (n = 10). Letters above bars and box plots (E-G) indicate statistically significant differences between samples as determined by Tukey’s HSD tests (P < 0.01).

MYC2, MYC3 and MYC4 are expressed basally in the root meristem (Fig 3) but insertion alleles in these genes do not show root growth alterations [20]. However, based on the scheme in Fig 1 it is possible that gain-of-function alleles of MYCs constitutively activate JA signalling. One such allele in *MYC3* is already known [29]. We extended the genetic screen used by [7] to search for new mutants that, under basal conditions, display ectopic expression of a secretable *JAZ10*_*pro*_ *-GUSPlus* ^*sec*^ (*JGP*) reporter. Unlike the weak *JGP* activity observed in the WT (Fig 4A) [7], one such mutant displayed basal *JGP* activity in the early differentiation zone of the primary root tip without reaching mature parts of the upper root (Fig 4B). The mutant segregated recessively from WT *MYC2* (S10 Fig) and was mapped by whole-genome sequencing to a G to A transition in position 493 of the *MYC2* gene causing a glutamate to lysine (E165K) substitution.

The relatively confined *JGP* expression of *myc2-322B* (Fig 4B) led us to hypothesize that a transcriptional repression mechanism is still able to inhibit the putative excessive MYC2^E165K^ activity in *myc2-322B*. Indeed, by removing the NINJA-dependent repression mechanism in *ninja-1 myc2-322B* double mutants, the effects on *JGP* activity became more remarkable, extending to a much wider domain in the primary root including the meristem (Fig 4C). To further assess the functionality of this novel *myc2* allele we tested its influence on JA-mediated gene expression. *myc2-322B* showed ~10 fold higher-than-WT *JAZ10* expression in 5-do roots (Fig 4D) as well as higher *JAZ10* accumulation in roots of cotyledon-wounded seedlings (S11 Fig). By contrast, neither basal *JGP* nor *JAZ10* expression differed from WT in aerial tissues (Fig 4B and S11 Fig). Consistent with our hypothesis that a NINJA-dependent repression mechanism blocks excessive JA responses in *myc2-322B*, basal *JAZ10* expression reached ~200 higher-than-WT levels in roots of *ninja myc2-322B* double mutants and intermediate *JAZ10* and *JGP* levels in double mutants homozygous for *ninja* and heterozygous for *myc2-322B* (*ninja myc2-322B/+*) (Fig 4D and S12 Fig). Although *myc2-322B* segregated recessively in the WT background, we found that it is a dosage-dependent, gain-of-function allele once it is released from NINJA-dependent repression mechanisms in specific zones of the primary root.

When grown in control conditions, *myc2-322B* had up to 20% shorter roots than WT (Fig 4E), associated with decreased meristem cell number and cell elongation in the differentiation zone (Fig 4F and 4G). Root growth was more strongly affected in *ninja myc2-322B* double mutants: primary root length in control conditions reached only 50% of the WT length, and both meristem cell number and cell elongation in the differentiation zone were markedly reduced, and were similar to those of WT treated with JA (Fig 4F and 4G).

Given that both MYC2 and MYC3 contribute to JA signalling in *ninja* mutants (Fig 3O), we extended our studies to the *atr2D* allele of *MYC3* in which a D94N missense mutation in the JAZ-interacting domain (JID) causes released repression from most JAZ proteins and activation of stress-responsive genes [19, 29, 30]. The *atr2D* mutant accumulated ~3 times higher-than-WT *JAZ10* transcript levels in roots of seedlings (S13 Fig) without altering root length (S14 Fig). Conversely, roots of *ninja-1 atr2D* double mutants accumulated ~100 fold higher *JAZ10* levels than WT but their length was similar to *ninja* mutants. *JAZ10* transcripts were ~30 fold higher-than-WT in roots of *myc2-322B atr2D* double mutants (S13 Fig) but this did not reduce root length beyond that in *myc2-322B* alone (S14 Fig).

### MYC2^E165K^ results in enhanced transcriptional activity and partial release from JAZ repression

**Fig 5:**
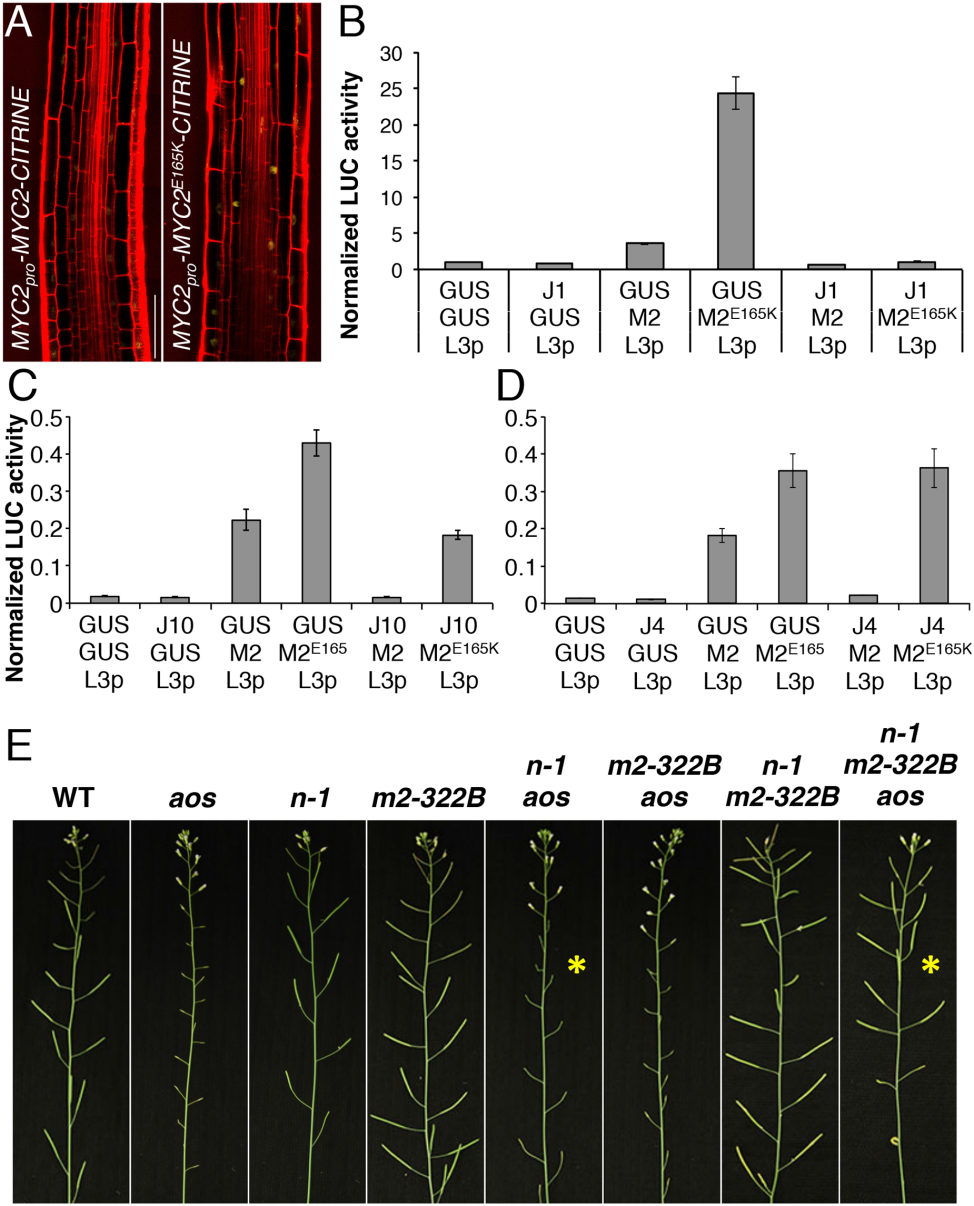
MYC2 ^E165K^ is a MYC2 gain-of-function allele. (A) Confocal microscopy images representing the expression pattern of functional *MYC2*_*pro*_ *-MYC2-CITRINE* and *MYC2*_*pro*_ *-MYC2* ^*E165K*^ *-CITRINE* fluorescent reporters (yellow) in the elongation zone of 5-do *jin1-7* roots stained with propidium iodide (red). Scale bar = 50 μm. (B-D) Transactivation of the *LOX3* promoter by transient expression of MYC2 or MYC2^E165K^ in the presence or absence of JAZ1, JAZ10 or JAZ4 repressors. Tobacco protoplasts were transfected with a *LOX3*_*pro*_ *-fLUC* (L3p) reporter construct, a *35S*_*pro*_ *-MYC2* (M2) or *35S*_*pro*_ *-MYC2* ^*E165K*^ (M2^*E165K*^) effector constructs in the presence or absence of *35S*_*pro*_ *-JAZ1* (JAZ1), *35S*_*pro*_ *-JAZ10* (J10) or *35S*_*pro*_ *-JAZ4* (JAZ4) constructs, and a *35S*_*pro*_ *-rLUC* normalization construct. The *35S*_*pro*_ *-GUS* (GUS) was used as control. Bars represent the means of 8 biological replicates (±SE) of normalized fLUC:rLUC activities. (E) Main inflorescences from 5 week-old plants of WT, *aos*, *ninja-1* (*n-1*), *myc2-322B* (*m2-322B*), *ninja-1 aos* (*n-1 aos*), *myc2-322B aos* (*m2-322B aos*), *ninja-1 myc2-322B* (*n-1 m2-322B*) and *ninja-1 myc2-322B aos* (*n-1 m2-322B aos*). Note the restored fertility in the *ninja-1 myc2-322B aos* triple mutant compared to the sterility of *ninja-1 aos* with a WT MYC2 protein (yellow asterisks).

The *myc2-322B* mutant presumably produces a MYC2 protein affected in the transcriptional activation domain (TAD, residues 149–188) responsible for recruiting the Mediator transcription initiation complex [15, 31]. Embedded in the TAD of MYC2, a stretch of acidic amino acids (MYC2^154–165^) constitutes the destruction element (DE) required for proteasome-dependent degradation and MYC2 functionality [31]. Deleting the entire DE resulted in a more stable MYC2 protein that could no longer induce the transcription of MYC2 regulated genes, such as *LOX2* [31]. The putative MYC2^E165K^ variant found in *myc2-322B* is altered in the last amino acid of the DE and could result in defective proteolysis and/or transcriptional activity. A functional MYC2^E165K^-CITRINE transgene driven by the endogenous *MYC2*_*pro*_ was expressed in the same root cells as WT MYC2-CITRINE but with a much stronger reporter signal (Fig 5A and S15 Fig). This apparently higher MYC2^E165K^ protein accumulation was not the result of increased transcripts as both basal and wound induced *MYC2* levels were the same between WT and *myc2-322B* mutant roots (S16 Fig). Furthermore, after inhibition of *de novo* protein synthesis with cycloheximide (CHX), MYC2^E165K^-CITRINE levels decreased over time, suggesting that the mutant protein is subjected to degradation just as WT MYC2-CITRINE (S17 Fig). Probably due to higher initial levels, MYC2^E165K^-CITRINE was still visible 60 min after CHX treatment, while MYC2-CITRINE had almost disappeared (S17 Fig). Concomitantly, we could also not detect aberrations in *LOX2* accumulation 1 h after wounding in *myc2-322B* (S18 Fig), implying that MYC2^E165K^ is functional.

We then assessed the transactivation capacity of MYC2^E165K^ and its regulation by JAZ repressors. In transient expression assays in tobacco protoplasts, MYC2^E165K^ had higher than WT activity in inducing the MYC2 responsive promoter of *LOX3* (*LOX3*_*pro*_) driving the expression of a *FIREFLY LUCIFERASE* (*fLUC*) reporter (Fig 5B–5D and S19 Fig). The transactivation of *LOX3*_*pro*_ by WT MYC2 was counteracted by co-expression with all 7 JAZ proteins tested (Fig 5B–5D and S19 Fig). However, the transactivation of *LOX3*_*pro*_ by the MYC2^E165K^ mutant protein was inhibited by co-expression with JAZ1 only, while it was less repressed by JAZ8, JAZ9 and JAZ10, and it failed to be inhibited by JAZ4, JAZ6 and JAZ12 (Fig 5B–5D and S19 Fig). We further compared the ability of WT MYC2 and MYC2^E165K^ to interact with 12 JAZ proteins in Yeast two-hybrid (Y2H) assays. As reported previously [19, 30], WT MYC2 was able to interact with all JAZs, except with JAZ7 (S20 Fig). On the other hand, MYC2^E165K^ was able to strongly interact only with JAZ1 in Y2H (S20 Fig). Thus, MYC2^E165K^ promotes the expression of early JA responsive genes as a consequence of both increased transactivation capacity and reduced inhibition by JAZ proteins. The gain-of-function behaviour of MYC2^E165K^ was then tested using an in planta genetic approach.

The *ninja-1* and *myc2-322B* mutations were introgressed into backgrounds that are fully male-sterile as a consequence of abolished JA production (*aos*) or signalling (*coi1-1*). Remarkably, when MYC2^E165K^ was liberated from NINJA-dependent repression, it was able to restore fertility of *aos* and of *coi1-1* mutants in *ninja-1 myc2-322B aos* and *ninja-1 myc2-322B coi1-1* combinations, whereas the WT copy of MYC2 failed to do so (Fig 5E and S21 Fig). Similarly to roots where basal *MYC2* expression was *aos*-independent (S6 Fig), *MYC2* transcript levels in stage 12 flowers were similar between WT and *aos*, whereas they were increased in the *ninja-1 myc2-322B aos* triple mutant (S22 Fig). Furthermore, we tested whether the restored fertility was a consequence of *MYB21* and *MYB24* induction, two TFs essential for male fertility whose expression is impaired in *aos* flowers [32]. In the *ninja-1 myc2-322B aos* triple mutant the expression of *MYB21* rose to WT levels and that of *MYB24* was intermediate between that of *aos* and WT. Conversely, the expression of both TFs was lower-than-WT in *ninja-1 aos* mutants with WT MYC2 (S22 Fig). The growth effects of MYC2^E165K^ are therefore not seedling specific, but extend into reproductive organs and adult-phase rosettes (S23 Fig). To consolidate this, we used a repetitive leaf-wounding assay that is known to cause a JA-dependent reduction in WT rosette growth [2, 4]. In this assay, the *myc2-322B* mutant was more sensitive than the WT to wound-induced growth reduction (S24 Fig). Finally, *myc2-322B* was more susceptible than WT when challenged with the generalist herbivore *Spodoptera littoralis* (S25 Fig).

### MYC2^E165K^ renders roots hypersensitive to exogenous JA

**Fig 6:**
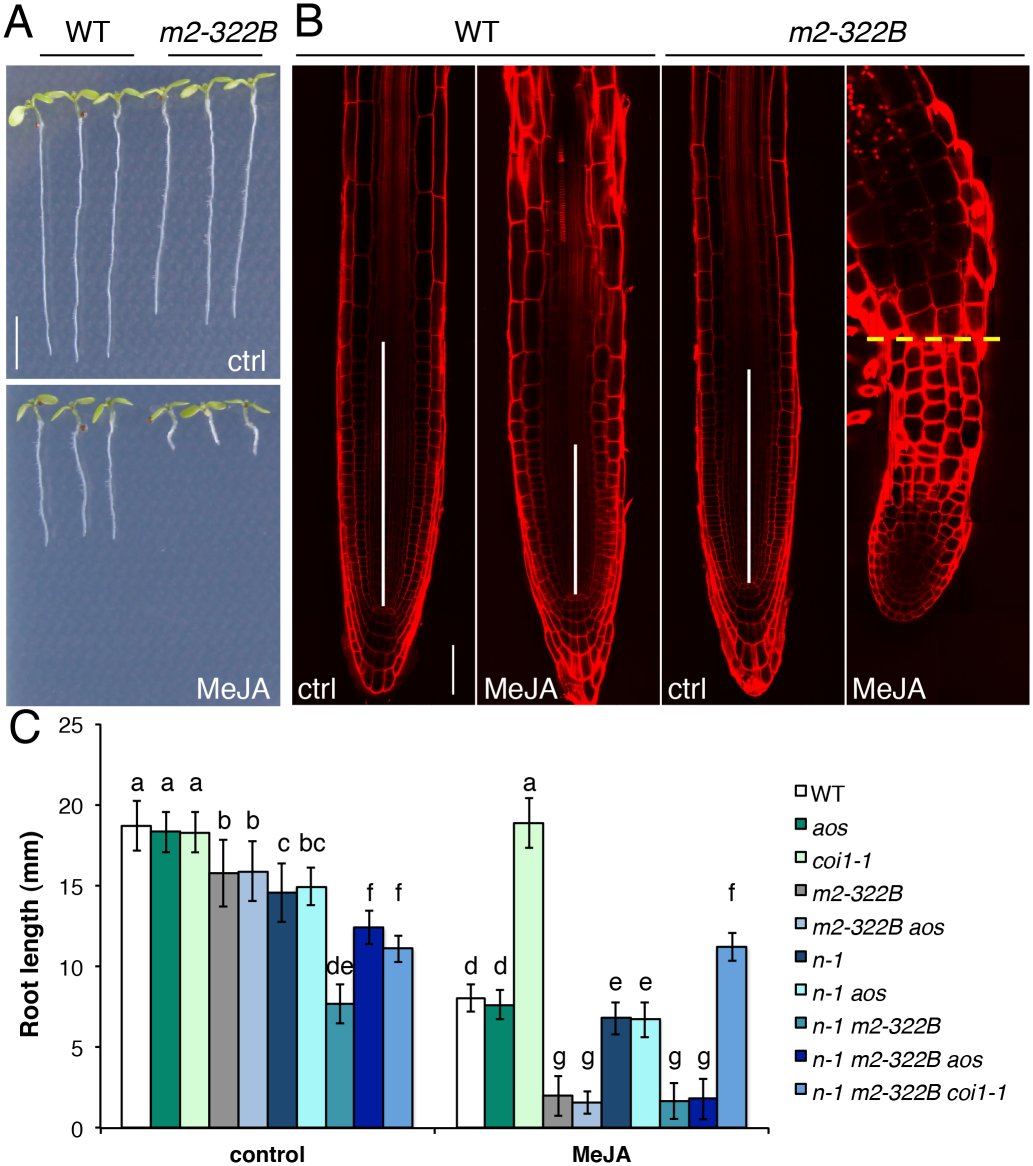
MYC2 ^E165K^ confers extreme hypersensitivity to exogenous JA. (A) Representative 7-do seedlings of WT and *myc2-322B* (*m2-322B*) mutants grown in control conditions (ctrl) or on media supplemented with 25 μM MeJA. Scale bar = 0.5 cm (B) Confocal microscopy images of propidium iodide stained primary root meristems of WT and *myc2-322B* 5-do seedlings grown in the absence (ctrl) or presence of 25 μM MeJA. Scale bar = 50 μm. Vertical white bars represent the root division zone and the horizontal yellow dashed line marks the root—hypocotyl boundary of *myc2-322B* grown in the presence of MeJA. (C) Root length of 7-do seedlings of the indicated genotype grown in the absence (control) or presence of 25 μM MeJA. *n-1* refers to *ninja-1*. Data shown are means (± SD) from 20–49 plants. Letters above bars indicate statistically significant differences between samples as determined by Tukey’s HSD test (P < 0.01).

Loss-of-function *myc2* alleles are partly insensitive to JA-mediated root growth inhibition while the overexpression of *MYC2* causes mild hypersensitivity [23] with 75% reduction in root length compared to the 50% reduction observed for the WT [20]. The *myc2-322B* mutant responded far more strongly to exogenous JA: its root length was up to 99% shorter in media supplemented with methyl JA (MeJA) compared to control conditions (Fig 6A–6C). The JA-mediated hypersensitivity phenotype was specific to *myc2-322B* and did not extend to the *atr2D* allele of MYC3 (S14 Fig) or to plants overexpressing a *MYC* ^*D105N*^ variant with diminished JAZ binding ability [30]. Moreover, all mutant combinations with *myc2-322B* displayed a hypersensitive phenotype in JA-mediated root growth inhibition assays (Fig 6C), with almost no measurable root meristem (Fig 6B). Triple mutant combinations of *ninja-1 myc2-322B aos* and *ninja-1 myc2 coi1-1* showed that the extreme root growth reduction constitutively observed in *ninja-1 myc2-322B* double mutants partly relies on de novo JA synthesis and signalling as triple mutants had intermediate root lengths between the *ninja- 1 myc2-322B* and *myc2-322B* mutants (Fig 6C). Consistently, *ninja-1 myc2-322B aos* was hypersensitive to MeJA treatment, while *ninja-1 myc2-322B coi1-1* was completely insensitive (Fig 6C).

## Discussion

Single wounding treatments or simulated herbivory in aerial organs transiently reduced root growth in *Arabidopsis* [33] and *Nicotiana attenuata* [34] although the effect was reported to occur independently of *aos* in *Arabidopsis*. Herein we show that, with a repetitive wounding treatment, a shoot-to-root signal reaches below ground organs and, through JA action, restricts growth by inhibiting both cell proliferation and cell elongation in the primary root tip. These two basic cellular processes influencing root growth are similarly affected when plants are grown in media supplemented with JA [31]. Likewise, JA was shown to control leaf growth by inhibiting cell proliferation and the onset of endoreduplication [35]. Our data reveal a primary function of endogenous JA in coordinating root growth after distal wounding and establish a robust assay with which to investigate this phenomenon.

### Distinct expression domains for JA signalling components in the root apex

NINJA is expressed ubiquitously in the primary root tip. This included not only cells in the differentiated parts of the root where a cell elongation defect and de-repressed JA signalling is observed in *ninja* mutants [7], but also cells of the root division zone where no apparent morphological defect is found in *ninja* mutants. Therefore, the lack of evident phenotypes in the root division zone of *ninja* mutants is not the result of differences in spatial distribution of the NINJA repressor, but likely the result of the presence of additional regulatory mechanisms such as direct recruitment of TPL by one or more NINJA-independent JAZs [11, 36]; direct recruitment of HISTONE DEACETYLASE 6 (HDA6) by JAZ1 to inhibit transcription [36]; MYC2 stability, turnover and phosphorylation [31]; and regulation of MYC-MED interactions to promote transcription. The enhanced phenotype of the *ninja-1 myc2-322B* mutant supports the control of MYC2 levels or activity as a cause of the restricted *ninja-1* phenotype.

Mutants in *NINJA* display shorter roots in the absence of JA due to a defect in cell elongation only [7]. Consistent with a specific role of NINJA in repressing TF(s) mediating JA signalling in the root [7], a whole class of up-regulated transcripts found in roots of *ninja* was typically JA-responsive, with no major trends from other hormonal pathways. Several studies showed that the hormone ethylene (ET) does not affect root meristem cell division activity but strongly reduces cell elongation [37–39]. Thus, the root growth defect observed in *ninja* mutants could potentially arise from activated ET signalling. However, transcriptome analysis of *ninja* roots did not identify ET-responsive transcripts or markers for enhanced ET signalling, such as *ACETYL-COA CARBOXYLASE 2* (*ACC2*), *ETHYLENE RESPONSE SENSOR 1* or *MYB59* [40]. Instead, the abundance of up-regulated transcripts of genes involved in secondary metabolism is probably the consequence of activated JA signalling in *ninja* roots. Likewise, the up-regulation of *ILL6* that is involved in JA-Ile turnover [41, 42] is indicative of induced JA responses in *ninja* roots.

TF(s) freed from NINJA-dependent repression in *ninja* mutants are expected to activate JA-signalling and root growth responses. However, double mutants between *ninja* and T-DNA alleles of *WRKY38* and *bHLH025*, two TFs that we found were overexpressed in *ninja* roots, did not suppress *ninja* root phenotypes. This indicated that, at least alone, these TFs do not strongly regulate JA signalling and root length in *ninja*. It is likely that several TF(s) act redundantly leading to ectopic *JAZ10* accumulation and root length reduction. Nonetheless, *ninja myc2 myc3* triple and *ninja myc2 myc3 myc4* quadruple mutants revealed that the combined activity of MYC2 and MYC3 (but not, or less so, that of MYC4), contributes to transcribe approximately half of the *JAZ10* transcripts overexpressed in *ninja* roots. MYC3 and MYC4 were shown to have *cis*-element binding specificity similar to that of MYC2 and to act additively with MYC2 in JA-mediated restriction of root growth [19]. These three TFs can form homo- and heterodimers in vivo to regulate gene expression [19]. It is conceivable that MYC2 and MYC3 form hetero-dimers and are subjected to NINJA-dependent repression specifically in epidermal and endodermal cells of the elongation and maturation zones where they are co-expressed. MYC2 and MYC4 could also form a similar module in the outer layers of the columella and lateral root cap where their expression domains coincide, while it is unlikely that MYC3 and MYC4 operate in such manner because their promoter activities did not overlap in any cell type of the root apex. The TF expression patterns also correlate with the stronger contribution of MYC2 and MYC3 to *JAZ10* expression in *ninja* roots, and the lack of a detectable effect of MYC4. Since loss-of-function of all three TFs did not suppress the root growth phenotype of *ninja* mutants, it is possible that the remaining JA signalling levels of triple and quadruple mutants are sufficient to repress root growth or that de-repressed JA signalling is not the direct cause of root growth reduction in *ninja*.

### *myc2-322B* is a gain-of-function *MYC2* mutant

In addition to *ninja* [7], *myc2-322B* represents a second JA signalling mutant with increased *JAZ10* expression in the root that is associated with reduced root growth. However, the phenotypes of *ninja* and *myc2-322B* are different: while *JGP* reporter expression coincides with reduced cell elongation in the root differentiation zone of *ninja* mutants, *JGP* expression in *myc2-322B* correlates only with reduced cell elongation in the root differentiation zone but not with the reduced cell proliferation observed in the root division zone. JA-induced MYC2 inhibits cell proliferation in the root division zone by directly repressing the expression of *PLETHORA* (*PLT*) genes that mediate auxin regulation of stem cell niche maintenance in the root division zone [25]. Because the regulatory function of TFs may depend on the cell-type specific network of interactions, the transcriptional outputs of MYC2 might differ in different cell-types and root areas: repression of *PLT* genes in the root division zone without activating *JAZ10* transcription and *JAZ10* activation in the root differentiation zone leading to compromised cell elongation. The different growth phenotypes of the mutants indicate that individual elements of the JA signalling pathway affect organ growth in different manners.

Although MYC2^E165K^ is mutated in the transcriptional activation domain (TAD), it lost the ability to interact with JAZ repressors with the exception of JAZ1, as indicated by Y2H assays. This suggests that, in addition to the previously defined JAZ-interacting domain (JID) of MYC2 [13, 14], the destruction element (DE) within the TAD of MYC2 [31] might influence JAZ binding. Accordingly, JAZ1 was able to fully repress MYC2^E165K^ transcriptional activity in protoplast transient expression assays; while JAZ8/-9/-10 were able to only partly repress MYC2^E165K^ transcriptional activity, and JAZ4-6-12 did not show repressor capacity on MYC2^E165K^. The observed repressor activity of JAZ8/-9/-10 on MYC2^E165K^ in plant protoplasts is likely due to the ability of JAZ proteins to form hetero-dimers among each other [43]. Specifically, JAZ1 was shown to interact with JAZ8, JAZ9 and JAZ10 [44, 45], suggesting that the repressor activity of JAZ8/-9/-10 observed in transient expression assays may rely on hetero-dimerization with a tobacco JAZ1 orthologue and consequent binding to MYC2^E165K^. The results emphasize the diversity among JAZ proteins in interacting with MYC2 and with one another [43]. Similarly to MYC2^E165K^, a recently identified MYC2^D105N^ allele mutated in the JID of MYC2 causes impaired protein interactions with most JAZ repressors [30]. However, the transactivation potential of MYC2^D105N^ did not differ from WT MYC2 in inducing *pLOX3-fLUC* in tobacco protoplasts [30], while that of MYC2^E165K^ from the *myc2-322B* mutant was much greater than WT MYC2. Since the TAD of MYC2 is also necessary for MED25 binding [15, 16], it also remains possible that the MYC2^E165K^ -MED25 interaction is altered in *myc2-322B*, favouring a more efficient docking of the Mediator complex to recruit RNA polymerase II and initiate gene transcription.

An alternative scenario is provided by our finding that MYC2^E165K^ seems to accumulate at higher levels than WT MYC2 in basal conditions. It is possible that MYC2^E165K^ is translated more rapidly than WT MYC2, as we did not detect differences in *MYC2* transcript levels between WT and *myc2-322B*. If this was the case, JAZ repression might be relieved if a higher number of MYC2^E165K^ molecules outcompetes the available number of JAZ repressors. This supports the control of MYC2 levels within strict limits as a regulatory layer of JA signalling. Such a mechanism is responsible for the strong effects of shade and light signalling in JA-regulated responses [46].

Finally, at the reproductive stage, *myc2-322B* in the appropriate background (*ninja-1 myc2-322B aos* or *ninja-1 myc2-322B coi1-1*) revealed that it is possible to recapitulate archetype hormone response phenotypes (in this case male fertility) in the absence of the hormone (JA) or of its receptor (COI1). These results also imply a putative role of MYC2 in male fertility as we found that basal *MYC2* expression in flowers is *aos*-independent. Thus, the gain-of-function *myc2-322B* allele released from NINJA-dependent repression could induce the expression of *MYB21* and *MYB24*, while WT MYC2 was unable to do so.

*Myc2-322B* represents a novel allele that may find many uses, for example in amplifying JA responses after mild stimulation. Moreover, this mutant will be useful for dissecting JA signalling events in both the adult and reproductive phases. In fact, the mutant rendered rosette leaves hypersensitive to wounding, although it displayed decreased resistance to a chewing herbivore relative to the WT. It is possible that the herbivore susceptibility phenotype of *myc2-322B* is a consequence of increased MYC2^E165K^ repressor activity on some defense genes (e.g. *PDF1*.*2*) [23]. Increasing JA signalling may not always lead to enhanced defence against herbivores.

### Layers of root growth regulation by JA signalling

The constitutive *JAZ10* expression and root length phenotypes in *myc2-322B* were relatively mild due to NINJA-dependent and -independent repression mechanisms, particularly the direct recruitment of HDA6 by JAZ1 [36]. Indeed, removing NINJA-dependent repression from MYC2^E165K^ in *ninja myc2-322B* double mutants led to much stronger phenotypes: extended *JGP* reporter activity along the whole root, 200 fold higher-than-WT root *JAZ10* expression and root length and cellular phenotypes analogous to those of the WT treated with JA. NINJA-dependent repression mechanisms probably inhibit the basal activity of MYC TFs expressed in the root, explaining the lack of morphological phenotypes of *myc* KO mutants. This was further confirmed in heterozygous *myc2-322B*/+ mutants that showed no defects in *JGP* reporter activity or root length phenotypes in the presence of functional NINJA, but that displayed increased *JAZ10* levels and reduced growth in a *ninja* background. A synergistic effect on *JAZ10* expression was also observed in a double mutant between *ninja-1* and a gain-of-function allele of MYC3, *atr2D*. However, the *ninja-1 atr2D* morphological root phenotypes did not differ from those of *ninja*, indicating that this mutant version of MYC3 induces JA signalling without affecting root growth.

It is likely that the activity of MYC2 is tightly controlled by yet additional NINJA-independent repression mechanisms because, except in *coi1-1* backgrounds, all mutant combinations with *myc2-322B* were hypersensitive to JA in root growth assays. These findings imply that such repression mechanisms are still able to partly repress basal JA responses in the *ninja myc2-322B* double mutant to allow root growth, and that they rely on JAZ repressors, particularly JAZ1, which are readily degraded in the presence of JA [13, 14]. NINJA-dependent and -independent mechanisms, together with tight controls on MYC2 levels or activity, represent multiple regulatory levels adopted to repress JA responses and guarantee normal root development in the absence of JA. However, MYCs are present in precise cell types and parts of the root, ready to activate JA responses in the event of a JA stimulus. Such a multilayered organization of JA signalling repression mechanisms also explains the lack of a JA-hypersensitive phenotype in *ninja* mutants. The NINJA-dependent repression mechanism requires docking onto JAZ repressors, thus JAZ stability is epistatic to NINJA-dependent repression and lack of a functional NINJA protein cannot render the roots more sensitive than WT in JA-mediated root growth inhibition assays.

### Conclusion

The study of plant development is generally carried out in the absence of physical injury. However, damage to plant tissues through herbivory and environmental insult is common if not omnipresent in nature. Under these conditions, the JA pathway, which has a low activity in unstressed vegetative tissues, is stimulated and imposes its activity on cell division and elongation. The resulting growth restriction can strongly impact plant productivity and is therefore of both fundamental and agronomic importance. Taking roots as a model, and using the simplified scheme for canonical JA signalling shown in Fig 1, we show that it is possible to manipulate regulatory layers in the JA pathway such that cell division and cell elongation can be constrained differentially. This type of approach may lead to future strategies to alter organ growth and, potentially, uncouple it from defense responses that occur when JA signalling is initiated. Our results underline the importance of cell-specific expression of JA signalling components and this will help generate new hypotheses. For example, of the three MYC TFs analysed herein, only MYC2 has been shown to be JA-inducible [19], as well as being expressed in the epidermis in the division zone in the root meristem (Fig 2F). This raises the intriguing possibility of MYC2 decoding very mild and localized JA-derived signals, resulting perhaps from microarthropod attack or during root penetration of soil where MYC2-expressing cells could be mildly squeezed resulting in local and transient JA production [47]. Such scenarios remain to be tested and may be facilitated with the use of *myc2-322B*, which amplifies JA-mediated growth responses.

## Materials and Methods

### Plant material and growth conditions

*Arabidopsis thaliana* accession Columbia (Col) was the WT line as well as the genetic background of previously described mutants and transgenic lines used in this study: *aos* [48], *coi1-1* [22], *JGP*, *ninja-1*, *ninja-2 and ninja-3* [7], *Jas9-VENUS* [26] *myc2* (*jin1-2*) and *jin1-7* [23], *atr2D* [29] and *myc3*, *myc4*, *myc23*, *myc24*, *myc34*, *myc234* [19]. The *myc2-322B* mutant allele was identified in a forward genetic screen for ectopic expression of a secretable *JAZ10*_*pro*_ *-GUSPlus* ^*sec*^ (*JGP*) reporter and was identified with whole-genome sequencing of bulk segregants, as described [7]. For experiments with *myc2-322B*, the WT control was the *JGP* reporter line shown to have the same root length, basal and wound induced *JAZ10* expression as Col [7]. The T-DNA lines of *wrky38* (SAIL_749_B02) and *bhlh025* (SALK_080900) were obtained from the Nottingham Arabidopsis Stock Centre.

After seed stratification for 2 d at 4°C, plants were grown at 21°C under 100 μE m^-2^ s^-1^ of light with photoperiod depending on the application (seedlings: 14 h light, 10 h dark; soil-grown plants for herbivory assays: 10 h light, 14 h dark; soil-grown plants for crosses and phenotyping: 24 h light). For seedling growth, seeds were surfaced-sterilized and grown on half-strength Murashige and Skoog solid medium (0.5X MS, 0.5 g/L MES hydrate, pH 5.7) supplemented with 0.7% agar (for horizontally-grown seedlings sawn on 200 μm pore size nylon mesh) or 0.85% agar (for vertically-grown seedlings), as described previously [7].

### Plant treatments

For repetitive wounding, cotyledons of vertically grown seedlings were pierced with a micro-needle (36 gauge beveled needle) in aseptic conditions under a stereomicroscope. Wounding started in the morning (7–8 am) of the third day after transfer to the phytotron (3-do seedlings) and was repeated every 12 h on alternate cotyledons, for a total of 5 wounds per seedling.

Single cotyledon wounding of seedling, MeJA treatments, root phenotypic measurements (total length of primary root and cellular measurements), herbivory assays with *S*. *littoralis* were performed as described [7].

### Histochemical detection of GUS activity in the primary root

GUS staining and histology of horizontally- grown entire seedlings were performed as in [7]. For GUS staining and sectioning of the primary root, vertically grown 5-do seedlings were carefully transferred to GUS staining solution (50 mM sodium phosphate buffer pH 7.0, 0.1% Triton X-100, 3 mM K4Fe(CN)_6_, 3 mM K3Fe(CN)_6_, 0.5 mg/ml X-Gluc) and incubated at 37°C in the dark for 2–4 h. For imaging the primary root tip, the reaction was stopped by replacing the staining solution with 50 mM sodium phosphate buffer pH 7.0. Roots were then immediately mounted in freshly prepared chloral hydrate: glycerol: water solution (8:2:1). For cross-sections of GUSPlus reporter lines, the staining solution was replaced with 15% EtOH in water for 30 min, followed by 30 min incubation in 30% EtOH at RT. Seedling were then transferred to a 1% agarose support, bunches of 10–15 roots were closely aligned at the root tip and submerged with 1% warm agarose. Hardened agarose blocks containing the aligned roots were excised in ~0.4 cm^3^ cubes that were dehydrated through an ethanol series (30%, 50%, 70%, 90% and twice absolute) under agitation for 30 min each at 4°C. Samples were embedded in Technovit 7100 resin (Haslab GmbH, Ostermundigen, Switzerland) according to the manufacturer’s instructions. Briefly, samples were vacuum infiltrated with infiltration solution: absolute EtOH (1: 1) for 2 h and with infiltration solution for 3 h. Finally, samples were hardened with embedding solution and hardened agarose blocks were aligned at the root tip, arranged in sectioning moulds and covered with additional embedding solution under anaerobic conditions. Samples were sectioned on a Leica RM2255 microtome using disposable Leica TC-65 blades into 6 μm sections and visualized with an upright Leica DM5500 microscope fitted with a DFC420 camera.

### Gene expression analyses

For qRT-PCR experiments of *JAZ10*, *MYC2*, *SUR1*, *VSP2* and *LOX2* 5-do seedlings were grown horizontally, separated in shoots and roots or kept intact and collected for basal and 1 h after cotyledon wounding expression analysis. For *PCNA1* and *CYCB1;1* expression, root samples were collected from 5-do vertically grown seedling subjected to repetitive cotyledon wounding. To determine the expression levels of *MYB21* and *MYB24*, stage 12 flowers (largest closed buds) from plants grown in continuous light were separated from the rest of the inflorescence and frozen in liquid N_2_. Each biological replica consisted of equivalent flower buds from 3–4 inflorescences of the same plant. For genotypes in which flower maturation was impaired (*aos*, *aos ninja-1*) or delayed (*aos myc2-322B ninja-1*), equivalent stage flowers were identified according to their position in the inflorescence stem. RNA and cDNA were prepared as in [49]. Quantitative RT-PCR was performed as described [50]. Primers for qRT-PCR have been previously reported: *JAZ10* (At5g13220) and *UBC21* (At5g25760) in [49], *SUR1* (At2g20610) in [51], *LOX2* (At3g45140) in [52], and *VSP2* (At5g24770) in [7]. To quantify other transcripts the following primers were used: *MYB21* (At3g27810, ggggaaacaggtggtcgaaa and tgcttgcagcttgatcgttg); *MYB24* (At5g40350, tctcgccaaatctgcaggac and ccacctatttccccattttgcat); *PCNA1* (At1g07370, tgggttacattcgttactac and atacaaaggaatctcacca), *CYCB1;1* (At4g37490, ccggaactgaatctgcttagg and gcgactcattagacttgttca) and *MYC2* (At1g32640, gtgcgggattagctggtaaa and atgcatcccaaacactcctc). RT-PCR in S9 Fig was performed with GoTaq DNA polymerase (Promega) on 5-do WT and T-DNA lines as indicated by the manufacturer, with the following primer pairs: for *WRKY38* cggtgcaagctatccgttat and ctttcactgccagatgacga, for *bHLH025* cgaccaacaatgatccctct and tgaaattcgacaaagcagacc.

For microarray analysis, seedlings were grown horizontally in aseptic conditions as indicated above. Total RNA was extracted from 5-do roots and purified with RNeasy Plant Mini Kit (Qiagen). Three biological replicates, each consisting of 120 roots, were performed per genotype. RNA quality was analysed with the Agilent 2100 Bioanalyzer (Agilent). RNA amplification and hybridization on Affymetrix ATH1 arrays were performed as described [53]. Microarray data analysis was performed in R. Raw data was normalized using GCRMA algorithm [54] to reduce variability between samples. Comparisons were performed on normalized data using a linear model [55]. Differentially expressed genes were identified using both a p-value cut-off, set to 0.05 after adjustment for false discovery rate [56], and fold change set to 2.

### Genotyping

Genotyping of T-DNA insertion lines was performed with the following oligonucleotides: tcttgtccggcaataaaaatg and aattaagtgagccgcgtactg for the WRKY38 WT allele (1.1 kb); tagcatctgaatttcataaccaatctcgatacac and aattaagtgagccgcgtactg for the *wrky38* T-DNA allele (600 bp); tagcgagatctttggttggtg and gctgttgcctctgaaaatctg for the bHLH025 WT allele (1.2 kb); attttgccgatttcggaac and gctgttgcctctgaaaatctg for the *bhlh025* T-DNA allele (700 bp). For selection of multiple mutant combinations from segregating populations *ninja-1* was amplified with ggaggatgagtcacggaaag and gggagctggactggtgagta primers and digested with *AciI* (WT = 359, 142 bp; mutant = 501 bp); *ninja-2* was amplified with tggtggttcttcttccaacc and gcaacaggttgtttgccttc primers and digested with *Hpy188I* (WT = 284, 209 bp; mutant = 284, 108, 101 bp); *ninja-3* was amplified with caacgggagacaacagcaac and tggcttgagagtttgatccg primers and digested with *TspRI* (WT = 302, 132, 2 bp; mutant = 436 bp); *myc2-322B* was amplified with gtcatcgaaaccaagaaaaacgatt and gagacggagatcgagttcgc primers and digested with *HinfI* (WT = 143, 25 bp; mutant = 168 bp); *atr2D* was amplified with caccacaacaaccacctcag and tgaagcagagaggcagagaag and digested with *BccI* (WT = 269, 162 bp; mutant = 431 bp).

### Transgenic lines

Promoters were amplified from WT genomic DNA with indicated oligonucleotides for *NINJA* (cggggtaccaatgctcatcctctgctgct and ttcccccccgggagcaaactctgagcaggtcaa, 2.7 kb), *MYC2* (cggggtacctcgtgtatttgtgtctgcatgt and ttcccccccgggtccataaaccggtgaccggtaa, 2.1 kb), *MYC3* (cggggtaccgcaaagaggatcgcttgaaa and ttcccccccggggtgaacatacgccggttgaaaag, 2 kb) and *MYC4* (cggggtaccacagtactaacgtttgatggaaac and ttcccccccgggaacagttctctgacgtagttataaaag, 1.5 kb) and cloned by restriction with *XmaI* and *KpnI* into a modified pUC57 [50] to create pEN-L4-promoter-R1 clones. Underlined sequences represent *XmaI* and *KpnI* sites. For promoter fusions, pEN-L4-promoter-R1- plasmids were recombined using the Gateway Cloning Technology with either pEN-L1-*GUSPlus*-L2 (non-secretable) or pEN-L1-*NLS3xVENUS*-L2 plasmids into pEDO097pFR7m24GW [50] to obtain pDEST-B4-promoter-B1-*GUSPlus*-B2 or pDEST-B4-promoter-B1-*NLS3xVENUS*-B2 clones. Coding DNA sequences (CDS) of *NINJA* (ggggacaagtttgtacaaaaaagcaggctgcatggacgatgataatgggctc and ggggaccactttgtacaagaaagctgggttggtgtgagctgacgctgcag), *MYC2* and *MYC2* ^*E165K*^ (ggggacaagtttgtacaaaaaagcaggctgcatgactgattaccggctaca and ggggaccactttgtacaagaaagctgggttaccgatttttgaaatcaaacttgc), *MYC3* (ggggacaagtttgtacaaaaaagcaggctgcatgaacggcacaacatcatc and ggggaccactttgtacaagaaagctgggttatagttttctccgactttcgtca), *MYC4* (ggggacaagtttgtacaaaaaagcaggctgcatgtctccgacgaatgttcaa and ggggaccactttgtacaagaaagctgggtttggacattctccaactttctcc) were amplified with oligonucleotides specified in parenthesis containing the appropriate *att* sites (underlined). CDSs of *NINJA* were amplified from WT cDNA, of *MYC2* ^*E165K*^ from *myc2-322B* genomic DNA, and of *MYC2*, *MYC3* and *MYC4* from WT genomic DNA. Amplification products were recombined into pDONR221 (Invitrogen) to produce pEN-L1-gene-L2 clones. To generate protein fusions under the control of endogenous promoters, pEN-L4-promoter-R1- plasmids were recombined with pEN-L1-CDS-L2 and pEN-R2-*CITRINE*-L3 plasmids into pB7m34gw by Multisite Gateway Technology to obtain pDEST-B4-promoter-B1-CDS-B2-*CITRINE*-B3 clones. All constructs were introduced into *Arabidopsis* backgrounds by floral dip Agrobacterium-mediated transformation. For promoter fusions, transformed seeds expressing red fluorescence protein in T_1_, T_2_ and T_3_ lines were selected by fluorescence microscopy, whereas for protein fusions, lines were selected on media containing DL-Phosphinothricin 40 μg/ml (Duchefa Biochemie B.V., Haarlem, The Netherlands). A minimum of two independent transgenic lines were used for each construct to perform experiments and verify reproducibility.

### Confocal microscopy

Confocal laser scanning microscopy was performed on a Zeiss LSM 700 confocal microscope with vertically grown 5-do seedlings. Roots were mounted in 0.5X MS with or without 30 μg/ml propidium iodide (Sigma). Excitation and detection windows were set as follows: VENUS 488 nm (dye EYFP), 490–555 nm (BP 490–555 filter); CITRINE 488 nm (dye Citr), 490–555 nm (BP 490–555 filter); propidium iodide 555 nm (dye PI), 615–700 nm (LP 615 filter). All images shown within one experiment were taken with identical settings. Image processing was done with FIJI (http://fiji.sc/Fiji).

### Transient expression assay in *Nicotiana tabacum* protoplasts

Transient expression assays were performed as described [57, 58]. Protoplasts were prepared from a Bright Yellow-2 tobacco cell culture and co-transfected with a reporter plasmid containing the firefly luciferase (*fLUC*) reporter gene driven by the *LOX3* promoter [59], a normalization construct expressing *Renilla* luciferase (rLUC) under control of the *35S* promoter [57] and effector constructs. Effector constructs were made by Gateway cloning of MYC2, MYC2^E165K^, JAZ1, JAZ4, JAZ6, JAZ8, JAZ9, JAZ10 and JAZ12 into the destination vector p2GW7 under control of the *35S* promoter. The p2GW7-GUS effector plasmid was used as mock [10]. For each transfection, 2 μg of each plasmid was used. After transfection, protoplasts were incubated overnight and then lysed; fLUC and rLUC activities were determined with the Dual-Luciferase reporter assay system (Promega). Variations in transfection efficiency and technical error were corrected by normalization of fLUC by rLUC activities. All transactivation assays were conducted in an automated experimental set-up with 8 biological replicates for each effector combination.

### Yeast two-hybrid analysis

Y2H analysis was performed as described [60]. Bait and prey were fused to the GAL4-AD or GAL4-BD via cloning into pGAL424gate or pGBT9gate (PSB, Ghent), respectively. The *Saccharomyces cerevisiae* PJ69-4A yeast strain [61] was co-transformed with bait and prey using the polyethylene glycol (PEG)/lithium acetate method. Transformants were selected on Synthetic Defined (SD) media lacking Leu and Trp (-2) (Clontech). Three individual colonies were grown overnight in liquid cultures (-2) at 30°C and 10-fold or 100-fold dilutions were dropped on control media (-2) and selective media lacking Leu, Trp and His (-3) (Clontech).

### Data access

Microarray data from this study have been submitted to the NCBI Gene Expression Omnibus (GEO; http://www.ncbi.nlm.nih.gov/geo/) under accession number GSE65840.

## Supporting Information

S1 Fig Schematic representation of a 5-do *Arabidopsis thaliana* primary root apex (half of a longitudinal section is shown). Arrowhead marks the end of the division zone and beginning of the elongation zone as depicted by the increase in cortex cell length; asterisks indicate the formation of lignified thickenings in the metaxylem and presence of root hairs in the epidermis marking the beginning of the maturation (differentiation) zone.
(PDF)

S2 Fig *Jas9-VENUS* reporter in the root meristem of 5-do WT seedlings grown in control conditions or subjected to repetitive cotyledon wounding. Samples were imaged 35 min after the last (5^th^) wound and represent merged overlays of the fluorescent (yellow) and the bright-field images. Scale bars = 100 μm.
(PDF)

S3 Fig *NINJA*_*pro*_ *-GUSPlus*, *MYC2*_*pro*_ *-GUSPlus*, *MYC3*_*pro*_ *-GUSPlus* and *MYC4*_*pro*_ *-GUSPlus* expression patterns determined by GUS staining in primary root meristems of 5-do WT seedlings. Weak GUS staining in cross-sections of *MYC2*_*pro*_ *-GUSPlus* is due to vacuolarization of maturing cells. Scale bars: entire seedlings = 0.5 mm; root meristems = 50 μm.
(PDF)

S4 Fig *NINJA*_*pro*_ *-NINJA-CITRINE*, *MYC2*_*pro*_ *-MYC2-CITRINE*, *MYC3*_*pro*_ *-MYC3-CITRINE* and *MYC4*_*pro*_ *-MYC4-CITRINE* expression patterns in primary root meristems of 5-do seedlings. Confocal microscopy images representing protein fusion expression patterns of the indicated fluorescent reporters (yellow) in 5-do roots stained with propidium iodide (red). Scale bar = 100 μm. Protein fusion functionalities for *NINJA*_*pro*_ *-NINJA-CITRINE*, *MYC2*_*pro*_ *-MYC2-CITRINE*, *MYC3*_*pro*_ *-MYC3-CITRINE* and *MYC4*_*pro*_ *-MYC4-CITRINE* are shown in S5 Fig.
(PDF)

S5 Fig Functionality assays of *NINJA*_*pro*_ *-NINJA-CITRINE*, *MYC2*_*pro*_ *-MYC2-CITRINE*, *MYC3*_*pro*_ *-MYC3-CITRINE and MYC4*_*pro*_ *-MYC4-CITRINE* constructs. (A) Root length of 8-do WT, *ninja-1* and three independent lines of *ninja-1* rescued with a *NINJA*_*pro*_ *-NINJA-CITRINE* transgene (42–3, 42–4, 42–11). Data shown are means (± SD) from 11–20 plants. Note the rescue of the *ninja-1* short root phenotype in the transgenic lines. (B) Root length of 7-do WT, *jin1-7* and two independent lines of *jin1-7* transformed with a *MYC2*_*pro*_ *-MYC2-CITRINE* construct (24–7, 24–11) grown on MS media supplemented with 25μM MeJA. Data shown are means (± SD) from 15–24 plants. Note the rescue of *jin1-7* root insensitivity to MeJA in the transgenic lines. (C) qRT-PCR of basal *SUR1* expression in 5-do seedlings of WT, *myc3*, two independent lines of *myc3* transformed with *MYC3*_*pro*_ *-MYC3-CITRINE* construct (44–4, 44–6), *myc4* and three independent lines of *myc4* transformed with *MYC4*_*pro*_ *-MYC4-CITRINE* construct (46–2, 46–7, 46–9). Transcript levels were normalized to those of *UBC21*. Bars represent the means of three biological replicates (±SD), each containing a pool of ~30 seedlings. Note the restoration of *SUR1* transcripts to WT levels in the transgenic lines.
(PDF)

S6 Fig Expression patterns of *NINJA*_*pro*_ *-NLS3xVENUS*, *MYC2*_*pro*_ *-NLS3xVENUS*, *MYC3*_*pro*_ *-NLS3xVENUS* and *MYC4*_*pro*_ *-NLS3xVENUS* florescent reporters in 5-do primary roots of *aos*. Images represent merged overlays of the fluorescent (yellow) and propidium iodide (red) stained roots. Scale bar = 50 m.
(PDF)

S7 Fig Root length of 7-do seedlings of *ninja-1 coi1-1* double mutants grown in control conditions or in the presence of 25 μM MeJA. Data shown are means (± SD) from 30–48 plants. Letters indicate statistically significant differences between pairs as determined by Tukey’s HSD test (P < 0.001).
(PDF)

S8 Fig qRT-PCR of *JAZ10* expression in roots of 5-do *ninja myc* mutant combinations 1 h after wounding one cotyledon. Genotypes are as follows: *ninja-1* (*n-1*), *ninja-2* (*n-2*), *myc2* (*m2*), *myc3* (*m3*), *myc4* (*m4*), *ninja-1 myc2* (*n-1 m2*), *ninja-1 myc3* (*n-1 m3*), *ninja-1 myc4* (*n-1 m4*), *myc2 myc3* (*m23*), *myc2 myc4* (*m24*), *myc3 myc4* (*m34*), *ninja-1 myc2 myc3* (*n-1 m23*), *ninja-1 myc2 myc4* (*n-1 m24*), *ninja-1 myc3 myc4* (*n-1 m34*), *myc2 myc3 myc4* (*m234*), *ninja-1 myc2 myc3 myc4* (*n-1 m234*), and *ninja-2 myc2 myc3 myc4* (*n-2 m234*). *JAZ10* transcript levels were normalized to those of *UBC21* and displayed relative to the expression of wounded WT controls that are set to 1 and indicated with a dashed line. Bars represent the means of three biological replicates (±SD), each containing a pool of ~60 roots. Complete qRT-PCR data are in S1 File.
(PDF)

S9 Fig *ninja* phenotypes are not suppressed in double mutants with T-DNA lines of up-regulated TFs found in the root transcriptome of *ninja-1*. (A) RT-PCR of full-length transcripts in WT and T-DNA lines of *wrky38* (SAIL_749_B02) and *bhlh025* (SALK_080900). The *UBC21* transcript (At5g25760) was used as the internal control. Note that the SALK_080900 T-DNA line is a promoter insertion that results in a hypomorphic allele with reduced *bHLH025* transcript level. (B) Root length in 7-do seedlings of the indicated genotype. Data shown are means (± SD) from 14–43 plants. Letters indicate statistically significant differences between pairs as determined by Tukey’s HSD test (P < 0.001). (C-D) qRT-PCR of basal *JAZ10* expression in indicated genotypes. *JAZ10* transcript levels were normalized to those of *UBC21* and displayed relative to the expression of unwounded WT controls (dashed lines), which are set to 1. Bars represent the means of three biological replicates (±SD), each containing a pool of organs from ~60 seedlings.
(PDF)

S10 Fig *myc2-322B* inheritance. Root details from GUS stained 5-do seedlings in uniform *JGP* backgrounds. The F_1_ progeny (*myc2-322B*/+) of a cross between *myc2-322* and the WT *JGP* line does not show ectopic *JGP* activity, whereas the F_1_ progeny (*myc2-322B*/-) between *myc2-322B* and a *myc2* null mutant (*jin1-2*) displays constitutive *JGP* activity, similar to that of *myc2-322B*.
(PDF)

S11 Fig qRT-PCR of *JAZ10* expression basally and 1 h after wounding aerial organs of 5-do *myc2-322B* lines. Abbreviations are as follows: *m2*-*322B* is *myc2-322B*, *n-1* is *ninja-1*, *n-2* is *ninja-2*. *JAZ10* transcript levels were normalized to those of *UBC21* and displayed relative to the expression of unwounded or wounded WT controls set to 1 (dashed lines). Bars represent the means of three biological replicates (±SD), each containing a pool of organs from ~60 seedlings. Complete qRT-PCR data are in S1 File.
(PDF)

S12 Fig *JGP* expression in 5-do seedlings and primary root meristem of *ninja-1 myc2-322B/+*. Note the weaker GUS staining in the *ninja-1 myc2-322B/+* heterozygous mutant compared to the *ninja-1 myc2-322B* double mutant in Fig 4C.
(PDF)

S13 Fig qRT-PCR of basal and 1 h after cotyledon wounding *JAZ10* expression in aerial and root organs of 5-do seedlings of mutant combinations with *atr2D*. Indicated genotypes: *m2 322B* is *myc2-322B*, *n-1* is *ninja-1*. *JAZ10* transcript levels were normalized to those of *UBC21* and displayed relative to the expression in the unwounded or wounded WT controls set to 1 (dashed lines). Bars represent the means of three biological replicates (±SD), each containing a pool of organs from ~60 seedlings. Complete qRT-PCR data are in S1 File.
(PDF)

S14 Fig Root length of 7-do seedlings of mutant combinations with *atr2D* grown in control conditions or in the presence of 25 μM MeJA. *m2-322B* refers to *myc2-322B* and *n-1* to *ninja-1*. Data shown are means (± SD) from 27–52 plants. Letters indicate statistically significant differences between pairs as determined by Tukey’s HSD test (P < 0.001).
(PDF)

S15 Fig Functionality of the MYC2^E165K^-CITRINE fusion protein. Root length of 7-do WT, *jin1-7*, *myc2-322B* and two independent lines of *jin1-7* transformed with a *MYC2*_*pro*_ *-MYC2* ^*E165K*^ *-CITRINE* construct (25–5, 25–8) grown on MS media supplemented with 25μM MeJA. Note the JA hypersensitive phenotype in the *jin1-7* transformed lines (for explanations, refer to Fig 6). Data shown are means (± SD) from 13–27 plants.
(PDF)

S16 Fig qRT-PCR of basal and 1 h after wounding one cotyledon *MYC2* expression in 5-do roots of WT and *myc2-322B*. Transcript levels were normalized to those of *UBC21* and displayed relative to the expression in the unwounded WT control. Bars represent the means of three biological replicates (±SD), each containing a pool of ~60 roots.
(PDF)

S17 Fig MYC2-CITRINE and MYC2^E165K^-CITRINE are degraded similarly following cycloheximide (CHX) treatment. 5-do *jin1-7* seedlings transformed with either *MYC2*_*pro*_ *-MYC2-CITRINE* or *MYC2*_*pro*_ *-MYC2* ^*E165K*^ *-CITRINE* were treated with 100 μM CHX for the indicated times, after which primary roots were stained with propidium iodide (red) and examined by confocal microscopy. Details of the vascular tissues in the elongation zone where the florescent (green) signal was more intense are shown. The experiment was repeated three times with two independent lines for each reporter. Scale bar = 50 μm. Note: because the expression of chimeric proteins is under the control of *MYC2* endogenous promoter, their expression level was too low to be detected in Western blots from 5-do seedlings. Moreover, although we analyzed 24 independent T_2_ lines, we failed to recover transgenic lines overexpressing MYC2^E165K^ protein under the *UBIQUITIN 10* promoter (At4g05320), suggesting that this protein version may be harmful to plants if expressed constitutively.
(PDF)

S18 Fig qRT-PCR of basal and 1 h after wounding one cotyledon *LOX2* expression in 5-do WT and *myc2-322B* entire seedlings. Transcript levels were normalized to those of *UBC21* and displayed relative to the expression in the unwounded WT control. Bars represent the means of three biological replicates (±SD), each containing a pool of ~40 seedlings.
(PDF)

S19 Fig Transactivation of the *LOX3* promoter by transient expression of MYC2 or MYC2^E165K^ in the presence or absence of JAZ6, JAZ8, JAZ9 and JAZ12 repressors. Tobacco protoplasts were transfected with a *LOX3*_*pro*_ *-fLUC* (LOX3p) reporter construct, a *35S*_*pro*_ *-MYC2* (M2) or *35S*_*pro*_ *-MYC2* ^*E165K*^ (M2^E165K^) effector constructs in the presence or absence of a *35S*_*pro*_ *-JAZ6* (JAZ6), *35S*_*pro*_ *-JAZ8* (JAZ8), *35S*_*pro*_ *-JAZ9* (JAZ9) or *35S*_*pro*_ *-JAZ12* (JAZ12) construct, and a *35S*_*pro*_ *-rLUC* normalization construct. The *35S*_*pro*_ *-GUS* (GUS) was used as control. Bars represent the means of 8 biological replicates (±SE) of normalized fLUC:rLUC activities.
(PDF)

S20 Fig WT MYC2 and MYC2^E165K^ interactions with 12 JAZ proteins in Yeast two-hybrid assays. Yeast cells co-transformed with prey (MYC2 or MYC2^E165K^) and baits (JAZ1-12) were selected and grown on synthetic defined media lacking Leu and Trp (-2) as transformation control and on selective media lacking Leu, Trp and His (-3) to test protein interactions.
(PDF)

S21 Fig Fertility phenotypes of mutant combinations with *myc2-322B*. Main inflorescences from 5 week-old plants of indicated genotypes. Note the lack of sterility in the *ninja-1 myc2-322B coi1-1* triple mutant.
(PDF)

S22 Fig qRT-PCR of *MYC2*, *MYB21 and MYB24* expression in floral organs of mutant combinations with *myc2-322B*. Transcript levels were normalized to those of *UBC21* and displayed relative to the expression in the WT controls. Bars represent the means of three biological replicates (±SD), each consisting of equivalent stage 12 flower buds from 3–4 inflorescences of the same plant.
(PDF)

S23 Fig Rosette phenotypes of mutant combinations with *myc2-322B*. Plants were grown in continuous days for 4 weeks. Scale (diameter of each pot) = 7cm.
(PDF)

S24 Fig *myc2-322B* is hypersensitive to repetitive wounding in adult rosettes. Rosette phenotypes of WT and *myc2-322B* plants grown in short days for 5 weeks. At the age of two weeks, plants were wounded 5 times on different leaves at 3-d intervals or gently touched on the same leaf (Mock). Leaves were treated in the following order: leaf 2 (L2), L4, L5, L6 and L8. Numbers below plants indicate rosette mean fresh weight ± SD, n = 6.
(PDF)

S25 Fig Box plot summary of *S*. *littoralis* larval weights after feeding for 10 d on adult *myc2-322B* plants. Medians and means are represented inside the boxes by solid and dotted lines respectively. Circles depict outlier data points beyond ±1.5X the interquartile range defined by the whiskers; numbers indicate n. Letters indicate statistically significant differences between pairs as determined by Tukey’s HSD test.
(PDF)

S1 Table Differentially expressed genes between 5-do *ninja-1* and WT roots. (DOCX)

S1 File qPCR raw data. (XLSX)
