# Supplementary figures and images for: Multilayered Organization of Jasmonate Signalling in the Regulation of Root Growth
Source: PLoS Genet. 2015 Jun 12;11(6):e1005300. doi: 10.1371/journal.pgen.1005300 (PMC4466561; doi:10.1371/journal.pgen.1005300)

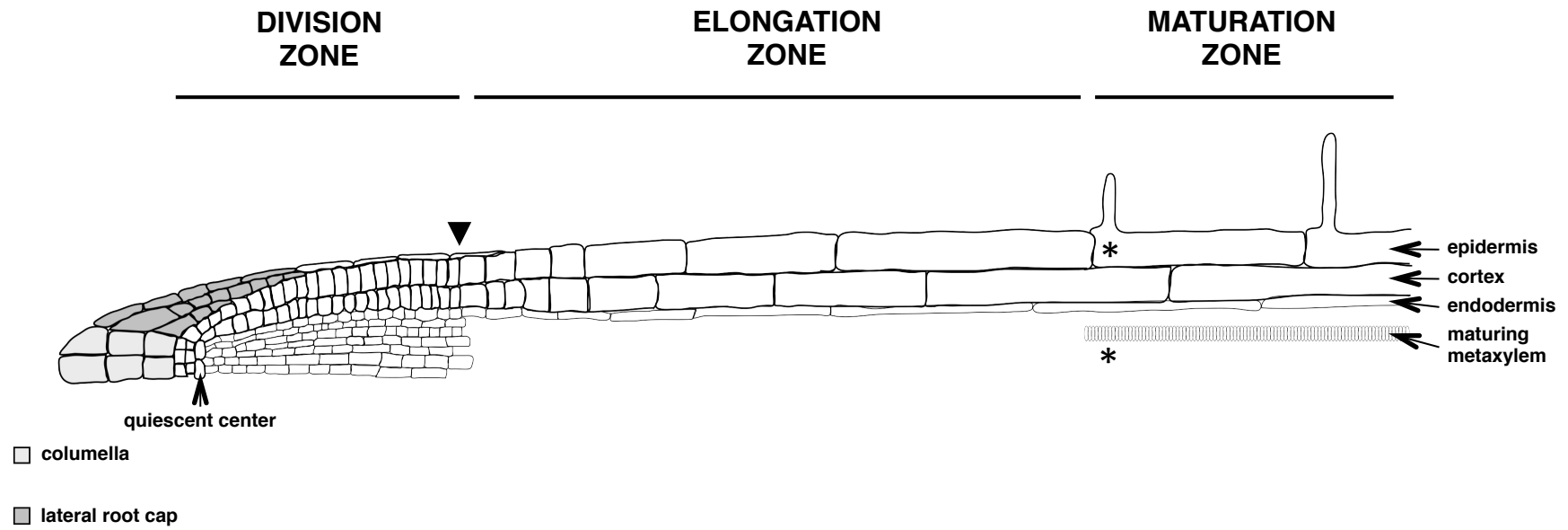

Supplement: S1 Fig — Arrowhead marks the end of the division zone and beginning of the elongation zone as depicted by the increase in cortex cell length; asterisks indicate the formation of lignified thickenings in the metaxylem and presence of root hairs in the epidermis marking the beginning of the maturation (differentiation) zone. (PDF) [file pgen.1005300.s001.pdf]

control

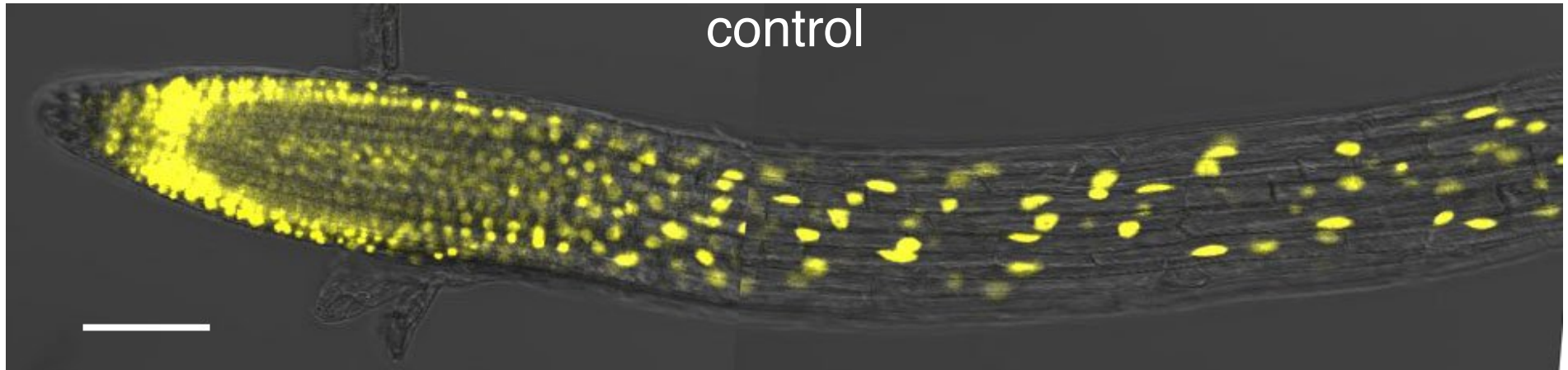

wounded

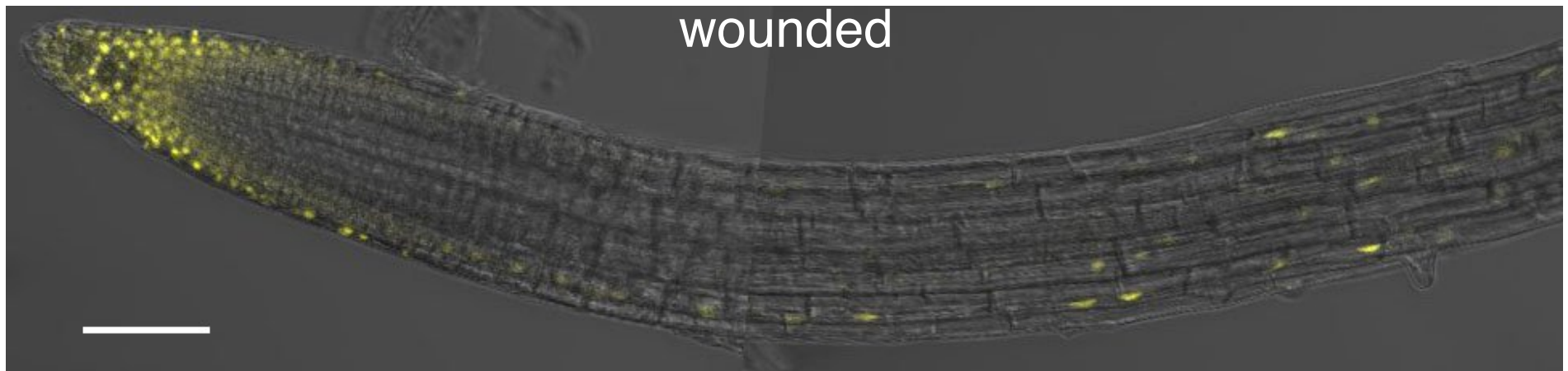

Supplement: S2 Fig — Samples were imaged 35 min after the last (5th) wound and represent merged overlays of the fluorescent (yellow) and the bright-field images. Scale bars = 100 μm. (PDF) [file pgen.1005300.s002.pdf]

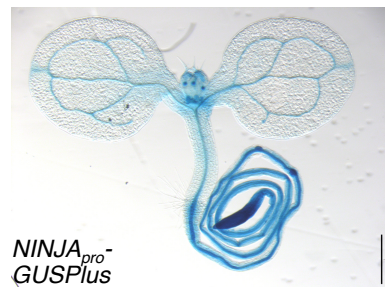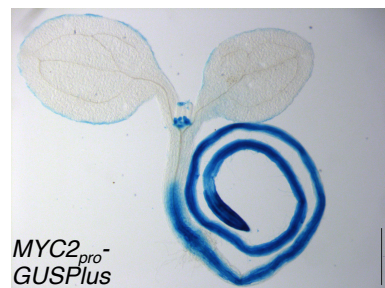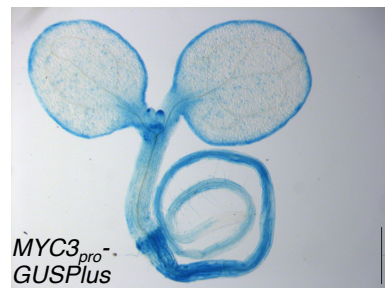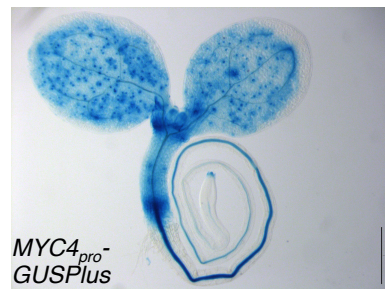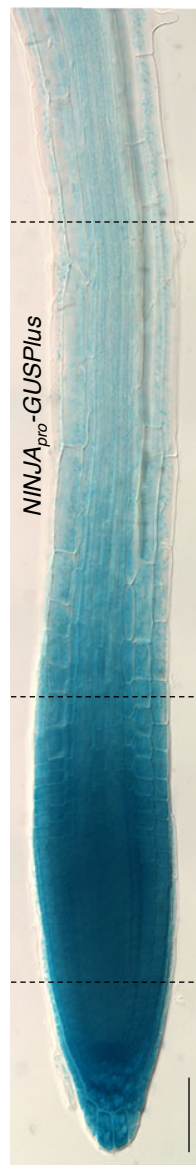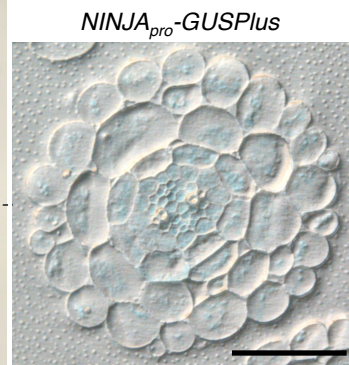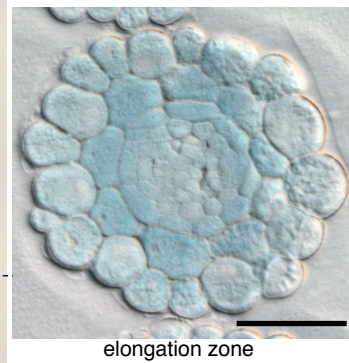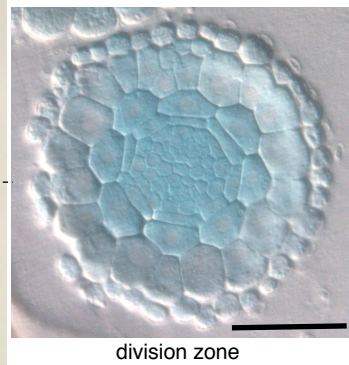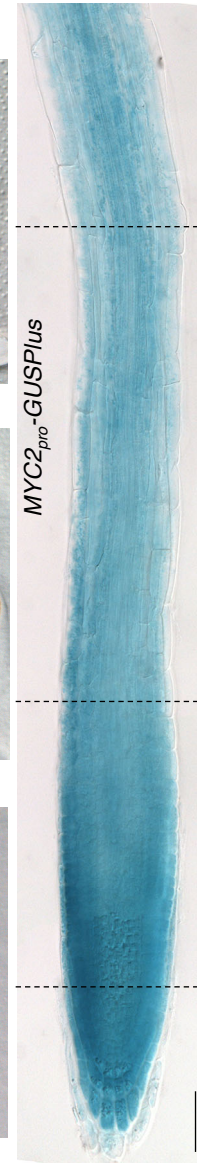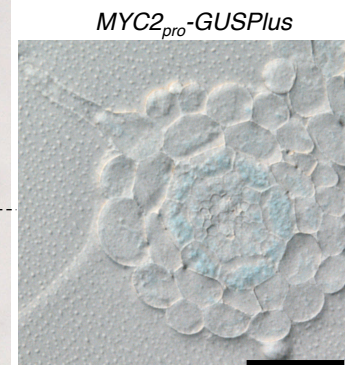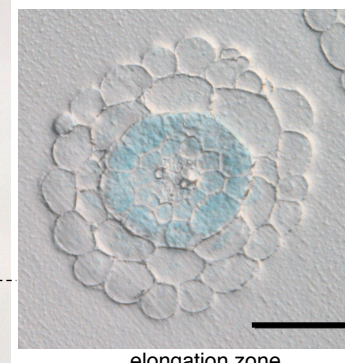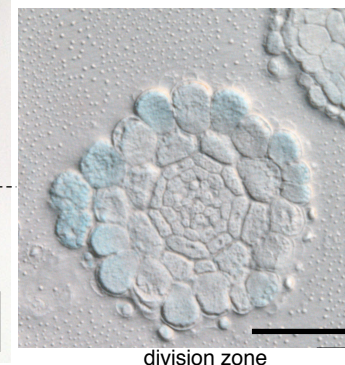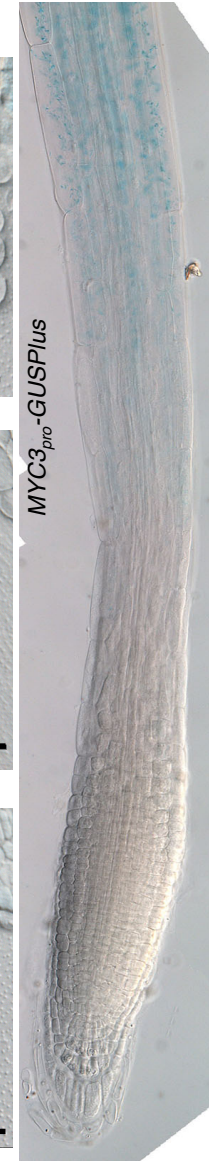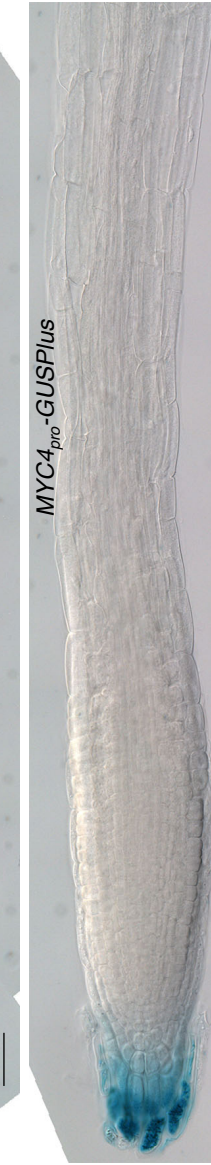

Supplement: S3 Fig — Weak GUS staining in cross-sections of MYC2 pro -GUSPlus is due to vacuolarization of maturing cells. Scale bars: entire seedlings = 0.5 mm; root meristems = 50 μm. (PDF) [file pgen.1005300.s003.pdf]

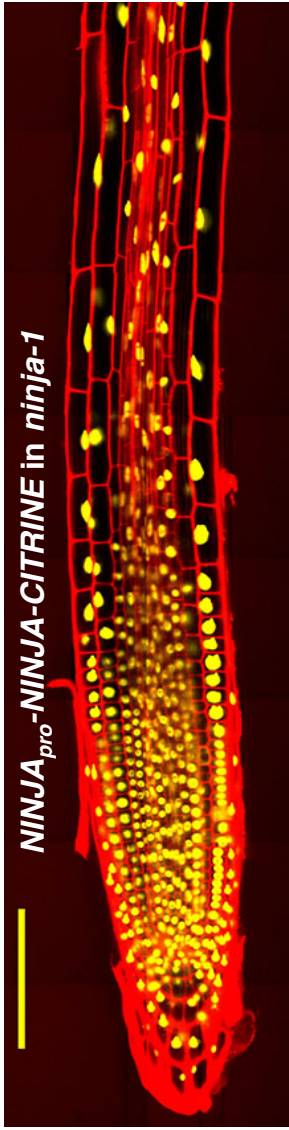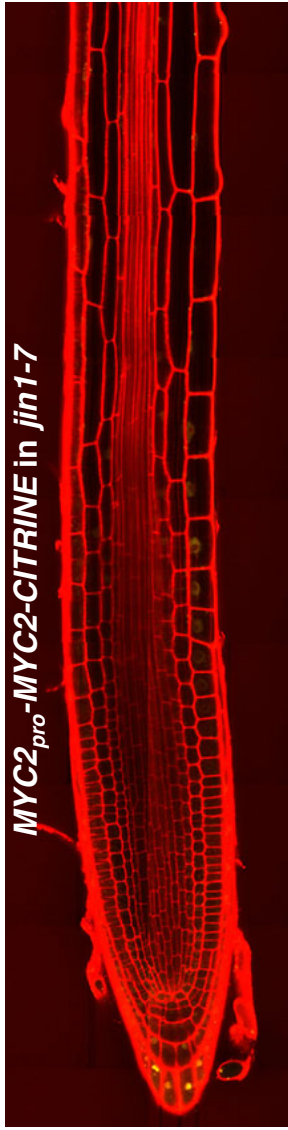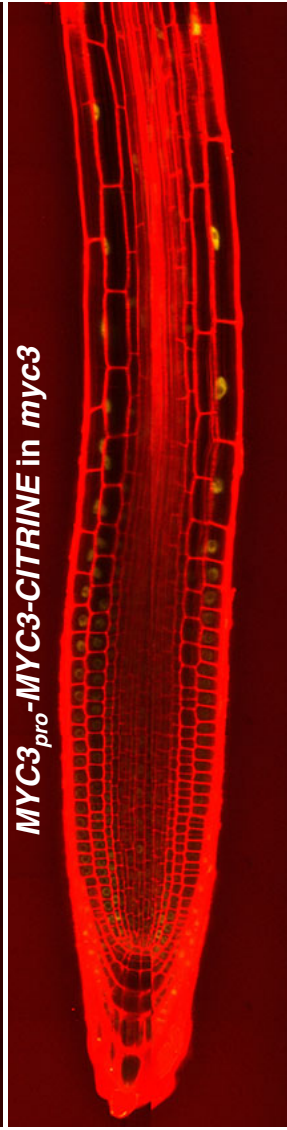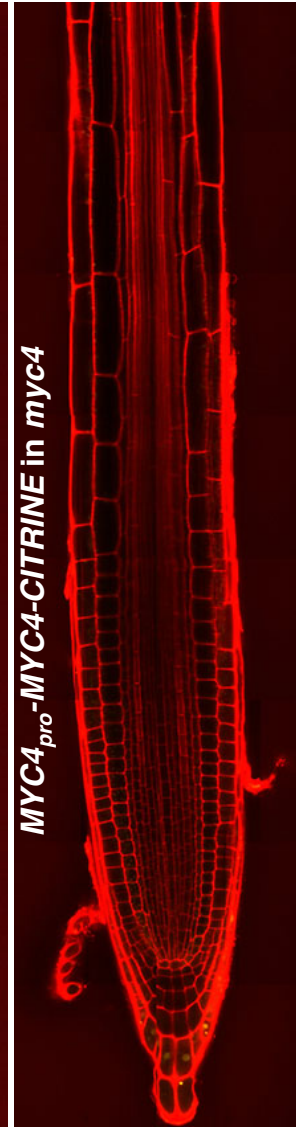

Supplement: S4 Fig — Confocal microscopy images representing protein fusion expression patterns of the indicated fluorescent reporters (yellow) in 5-do roots stained with propidium iodide (red). Scale bar = 100 μm. Protein fusion functionalities for NINJA pro -NINJA-CITRINE, MYC2 pro -MYC2-CITRINE, MYC3 pro -MYC3-CITRINE and MYC4 pro -MYC4-CITRINE are shown in S5 Fig. (PDF) [file pgen.1005300.s004.pdf]

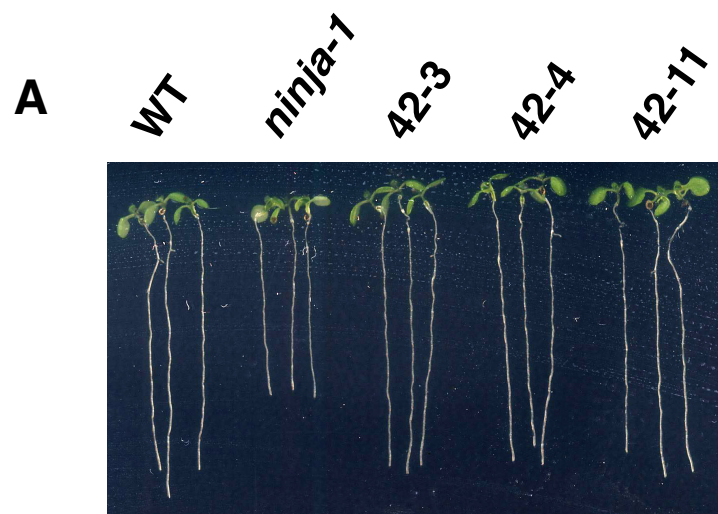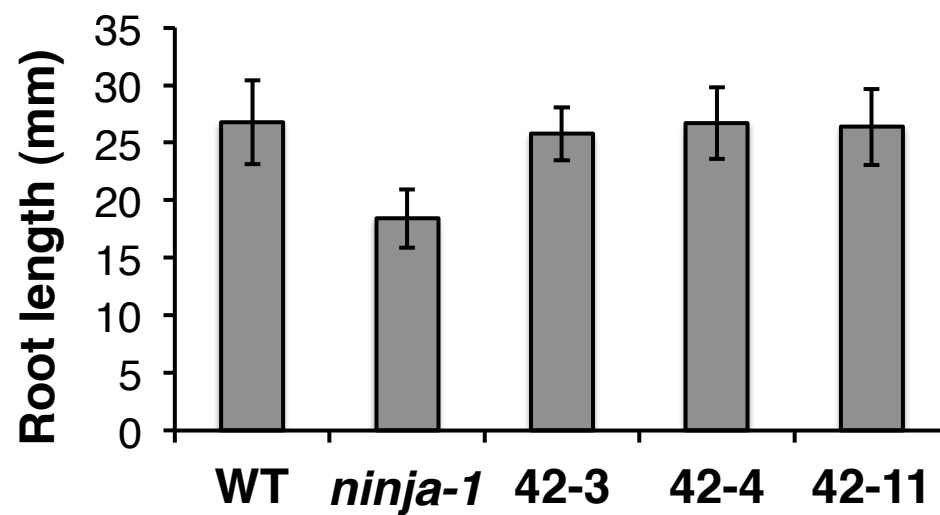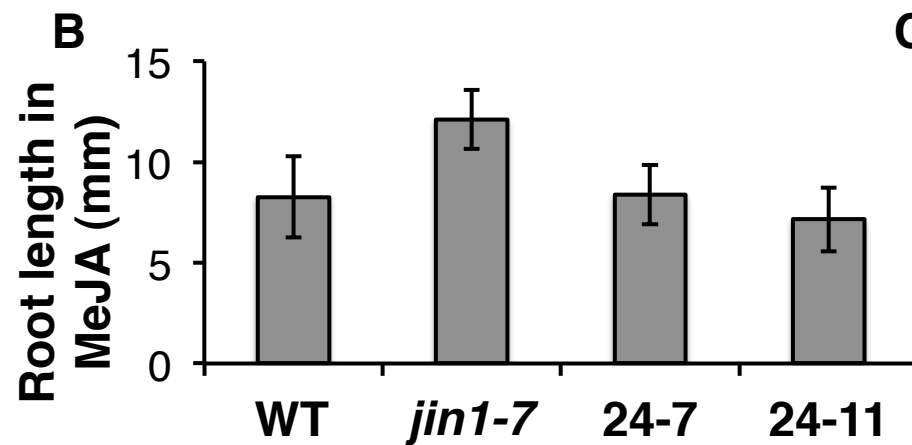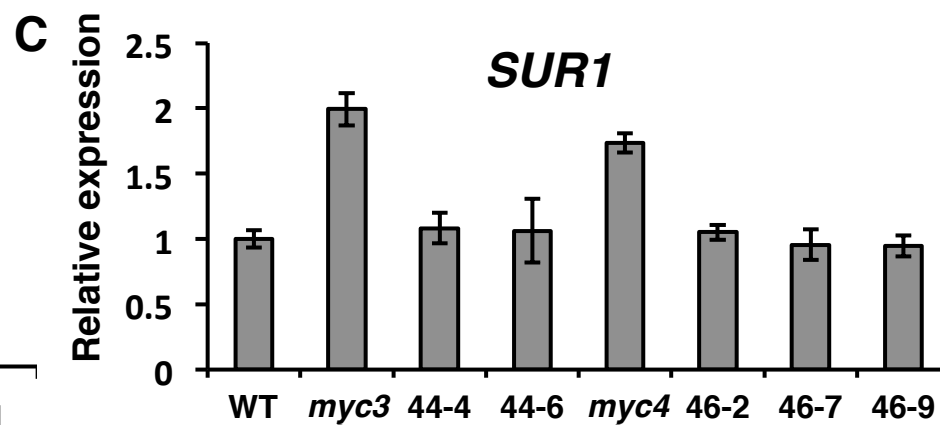

Supplement: S5 Fig — (A) Root length of 8-do WT, ninja-1 and three independent lines of ninja-1 rescued with a NINJA pro -NINJA-CITRINE transgene (42–3, 42–4, 42–11). Data shown are means (± SD) from 11–20 plants. Note the rescue of the ninja-1 short root phenotype in the transgenic lines. (B) Root length of 7-do WT, jin1-7 and two independent lines of jin1-7 transformed with a MYC2 pro -MYC2-CITRINE construct (24–7, 24–11) grown on MS media supplemented with 25μM MeJA. Data shown are means (± SD) from 15–24 plants. Note the rescue of jin1-7 root insensitivity to MeJA in the transgenic lines. (C) qRT-PCR of basal SUR1 expression in 5-do seedlings of WT, myc3, two independent lines of myc3 transformed with MYC3 pro -MYC3-CITRINE construct (44–4, 44–6), myc4 and three independent lines of myc4 transformed with MYC4 pro -MYC4-CITRINE construct (46–2, 46–7, 46–9). Transcript levels were normalized to those of UBC21. Bars represent the means of three biological replicates (±SD), each containing a pool of ~30 seedlings. Note the restoration of SUR1 transcripts to WT levels in the transgenic lines. (PDF) [file pgen.1005300.s005.pdf]

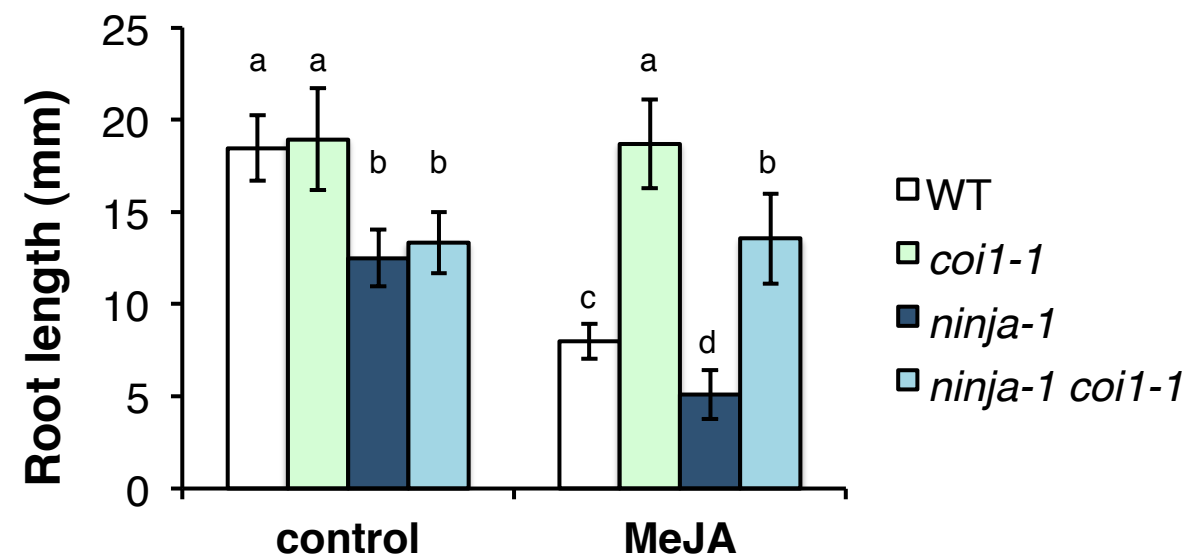

Supplement: S7 Fig — Data shown are means (± SD) from 30–48 plants. Letters indicate statistically significant differences between pairs as determined by Tukey’s HSD test (P < 0.001). (PDF) [file pgen.1005300.s007.pdf]

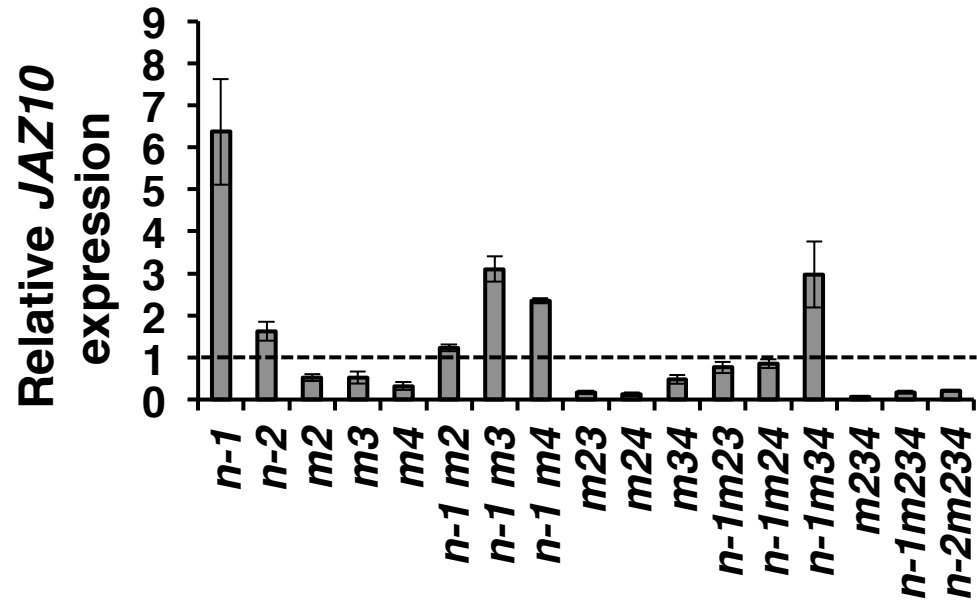

Supplement: S8 Fig — Genotypes are as follows: ninja-1 (n-1), ninja-2 (n-2), myc2 (m2), myc3 (m3), myc4 (m4), ninja-1 myc2 (n-1 m2), ninja-1 myc3 (n-1 m3), ninja-1 myc4 (n-1 m4), myc2 myc3 (m23), myc2 myc4 (m24), myc3 myc4 (m34), ninja-1 myc2 myc3 (n-1 m23), ninja-1 myc2 myc4 (n-1 m24), ninja-1 myc3 myc4 (n-1 m34), myc2 myc3 myc4 (m234), ninja-1 myc2 myc3 myc4 (n-1 m234), and ninja-2 myc2 myc3 myc4 (n-2 m234). JAZ10 transcript levels were normalized to those of UBC21 and displayed relative to the expression of wounded WT controls that are set to 1 and indicated with a dashed line. Bars represent the means of three biological replicates (±SD), each containing a pool of ~60 roots. Complete qRT-PCR data are in S1 File. (PDF) [file pgen.1005300.s008.pdf]

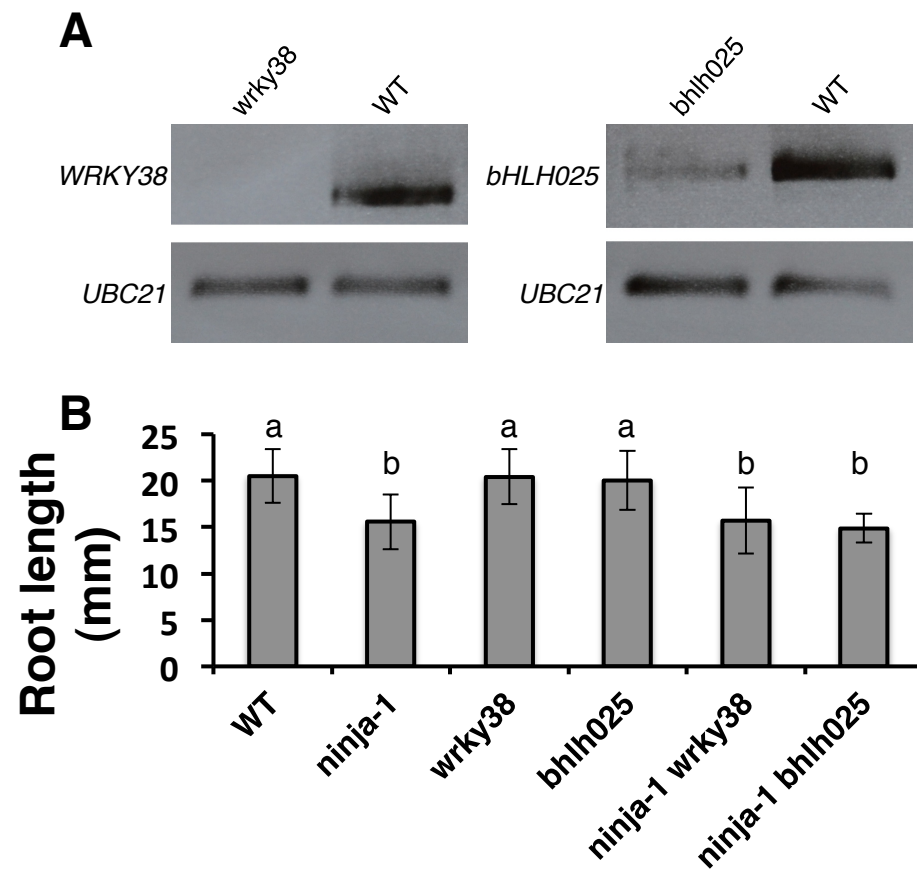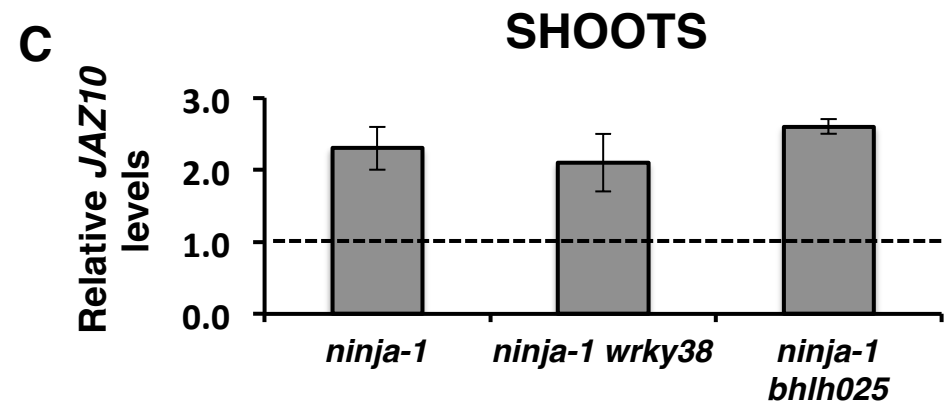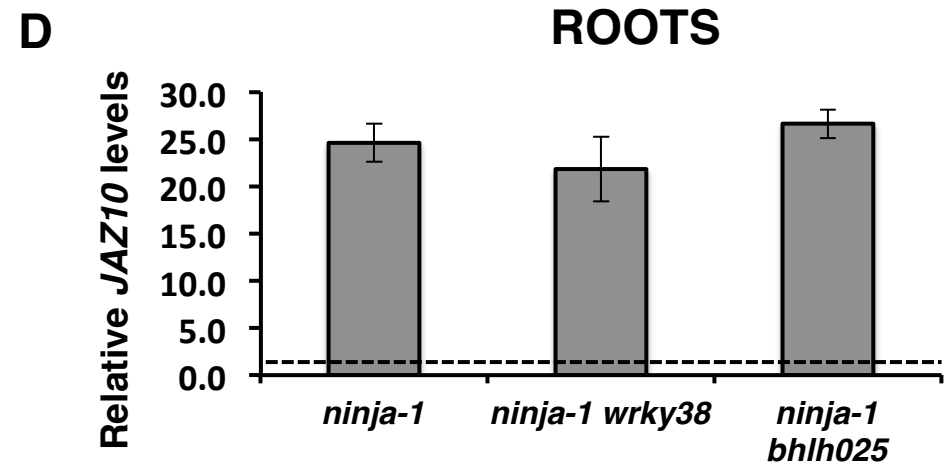

Supplement: S9 Fig — (A) RT-PCR of full-length transcripts in WT and T-DNA lines of wrky38 (SAIL_749_B02) and bhlh025 (SALK_080900). The UBC21 transcript (At5g25760) was used as the internal control. Note that the SALK_080900 T-DNA line is a promoter insertion that results in a hypomorphic allele with reduced bHLH025 transcript level. (B) Root length in 7-do seedlings of the indicated genotype. Data shown are means (± SD) from 14–43 plants. Letters indicate statistically significant differences between pairs as determined by Tukey’s HSD test (P < 0.001). (C-D) qRT-PCR of basal JAZ10 expression in indicated genotypes. JAZ10 transcript levels were normalized to those of UBC21 and displayed relative to the expression of unwounded WT controls (dashed lines), which are set to 1. Bars represent the means of three biological replicates (±SD), each containing a pool of organs from ~60 seedlings. (PDF) [file pgen.1005300.s009.pdf]

**Parental  
lines**

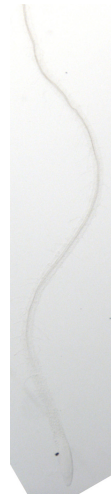

*JGP*

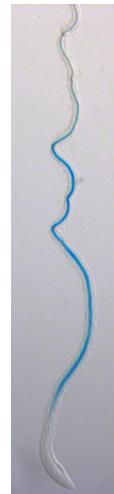

*JGP in  
myc2-322B*

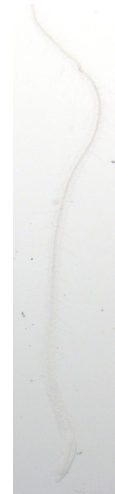

*JGP in  
jin1-2*

**F<sub>1</sub>**

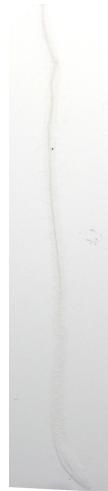

*myc2-322B/+*

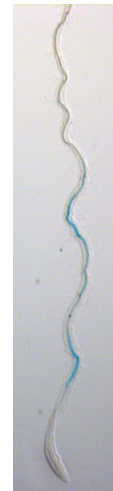

*myc2-322B/-*

Supplement: S10 Fig — Root details from GUS stained 5-do seedlings in uniform JGP backgrounds. The F1 progeny (myc2-322B/+) of a cross between myc2-322 and the WT JGP line does not show ectopic JGP activity, whereas the F1 progeny (myc2-322B/-) between myc2-322B and a myc2 null mutant (jin1-2) displays constitutive JGP activity, similar to that of myc2-322B. (PDF) [file pgen.1005300.s010.pdf]

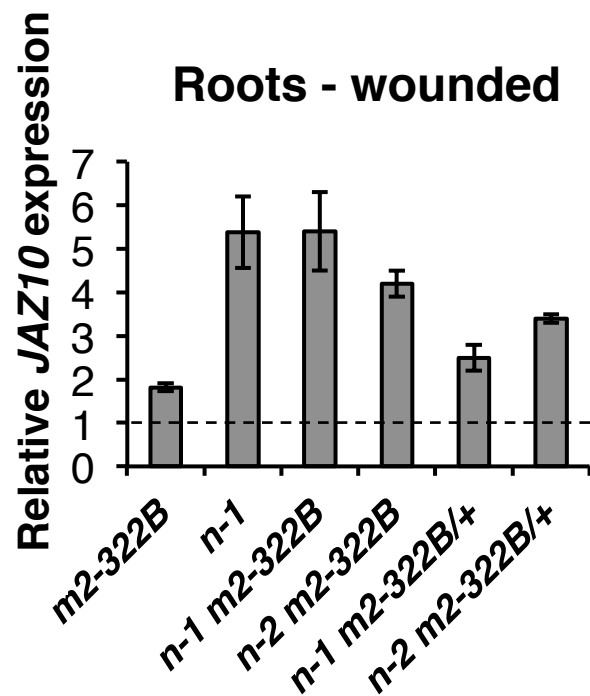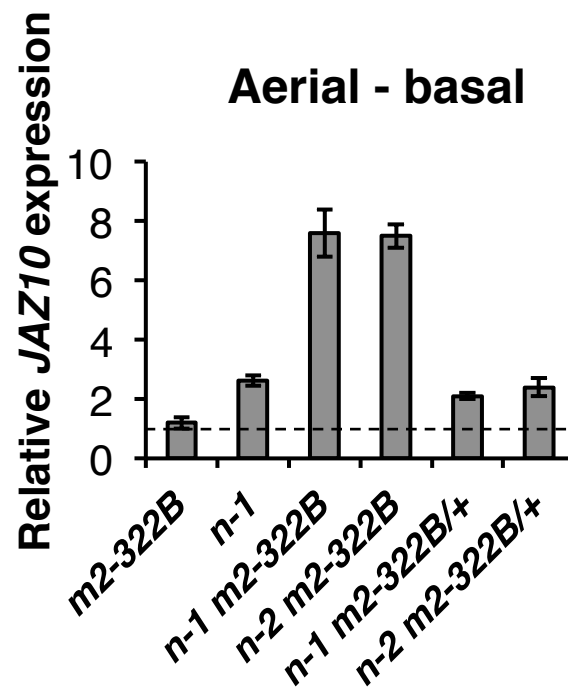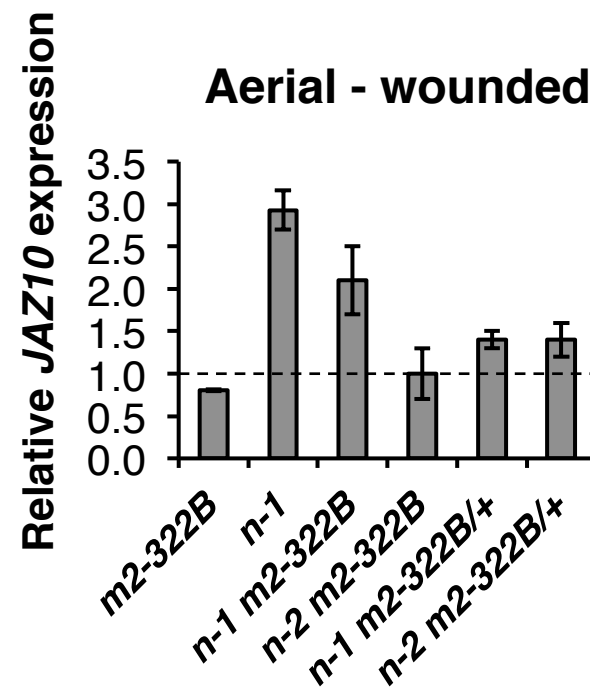

Supplement: S11 Fig — Abbreviations are as follows: m2-322B is myc2-322B, n-1 is ninja-1, n-2 is ninja-2. JAZ10 transcript levels were normalized to those of UBC21 and displayed relative to the expression of unwounded or wounded WT controls set to 1 (dashed lines). Bars represent the means of three biological replicates (±SD), each containing a pool of organs from ~60 seedlings. Complete qRT-PCR data are in S1 File. (PDF) [file pgen.1005300.s011.pdf]

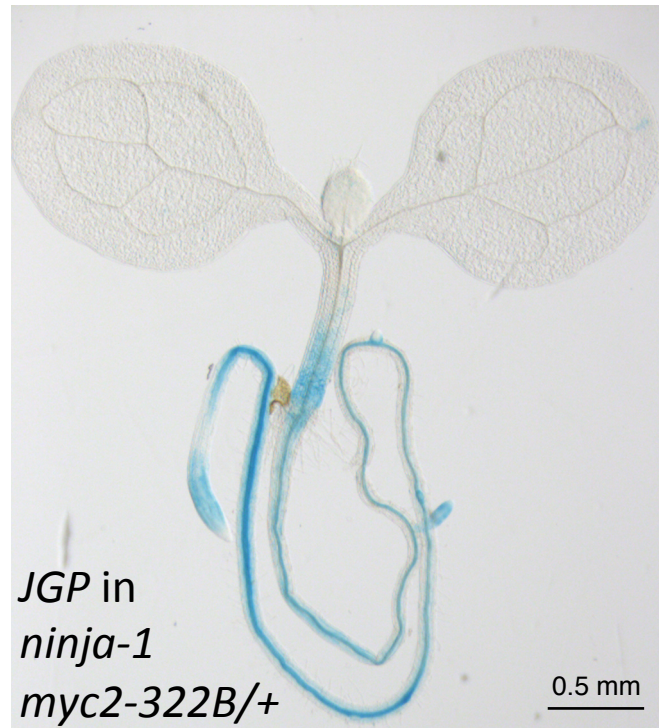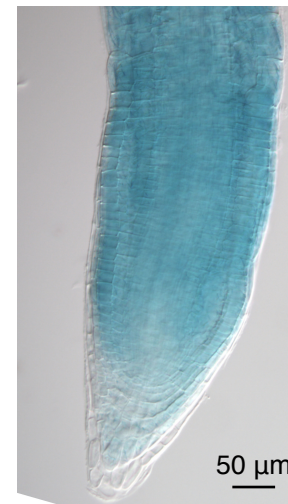

*JGP in  
ninja-1  
myc2-322B*

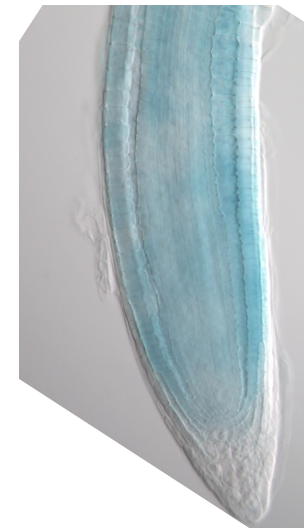

*JGP in  
ninja-1  
myc2-322B/+*

Supplement: S12 Fig — Note the weaker GUS staining in the ninja-1 myc2-322B/+ heterozygous mutant compared to the ninja-1 myc2-322B double mutant in Fig 4C. (PDF) [file pgen.1005300.s012.pdf]

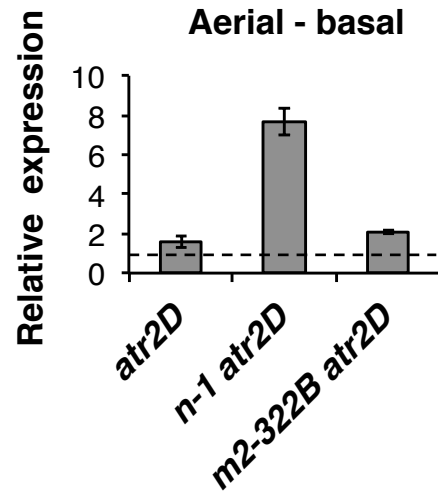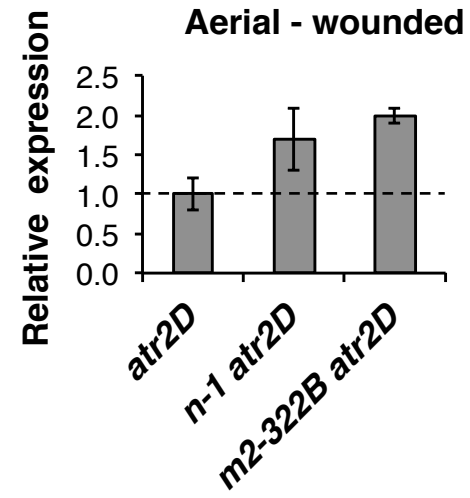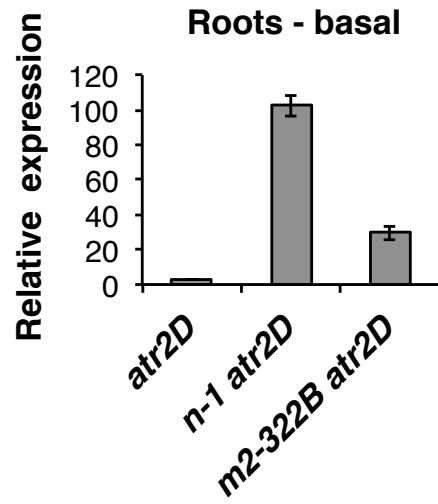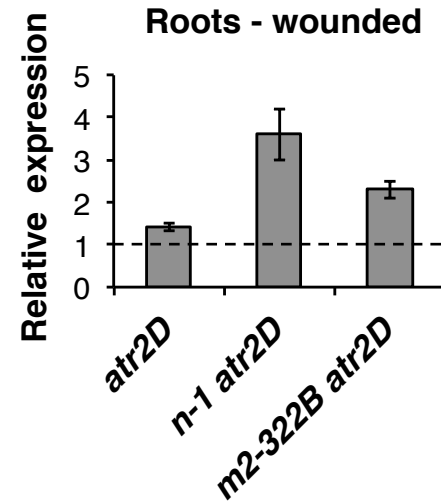

Supplement: S13 Fig — Indicated genotypes: m2 322B is myc2-322B, n-1 is ninja-1. JAZ10 transcript levels were normalized to those of UBC21 and displayed relative to the expression in the unwounded or wounded WT controls set to 1 (dashed lines). Bars represent the means of three biological replicates (±SD), each containing a pool of organs from ~60 seedlings. Complete qRT-PCR data are in S1 File. (PDF) [file pgen.1005300.s013.pdf]

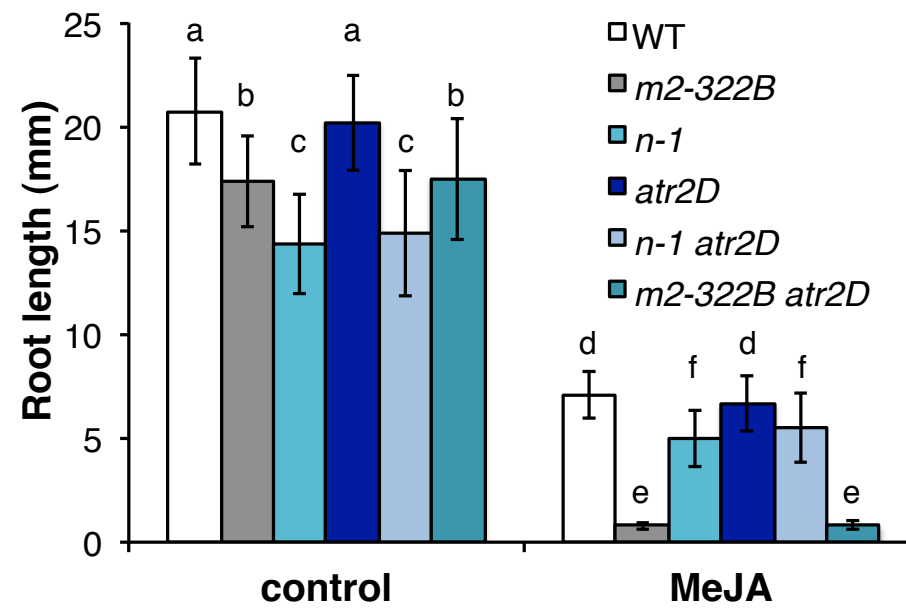

Supplement: S14 Fig — m2-322B refers to myc2-322B and n-1 to ninja-1. Data shown are means (± SD) from 27–52 plants. Letters indicate statistically significant differences between pairs as determined by Tukey’s HSD test (P < 0.001). (PDF) [file pgen.1005300.s014.pdf]

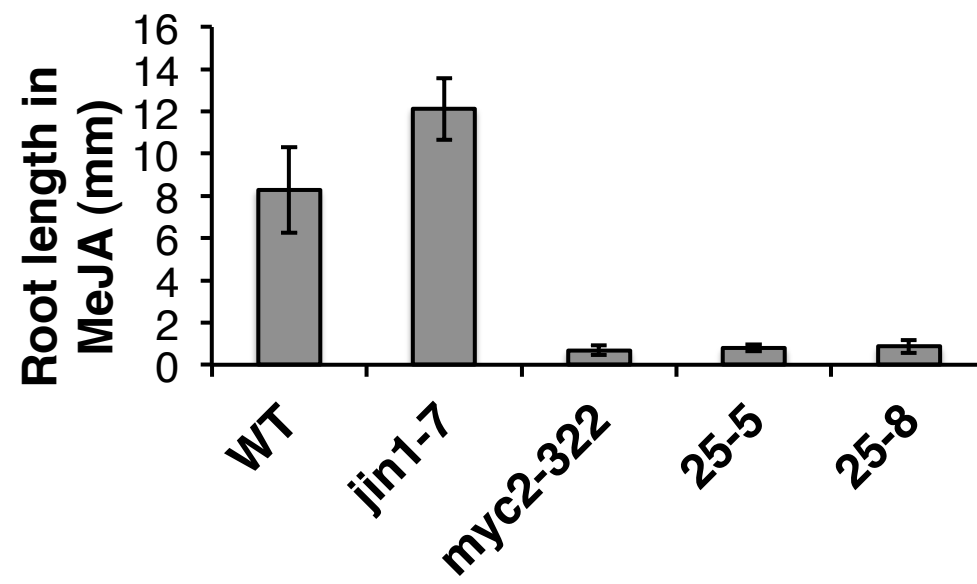

Supplement: S15 Fig — Root length of 7-do WT, jin1-7, myc2-322B and two independent lines of jin1-7 transformed with a MYC2 pro -MYC2 E165K -CITRINE construct (25–5, 25–8) grown on MS media supplemented with 25μM MeJA. Note the JA hypersensitive phenotype in the jin1-7 transformed lines (for explanations, refer to Fig 6). Data shown are means (± SD) from 13–27 plants. (PDF) [file pgen.1005300.s015.pdf]

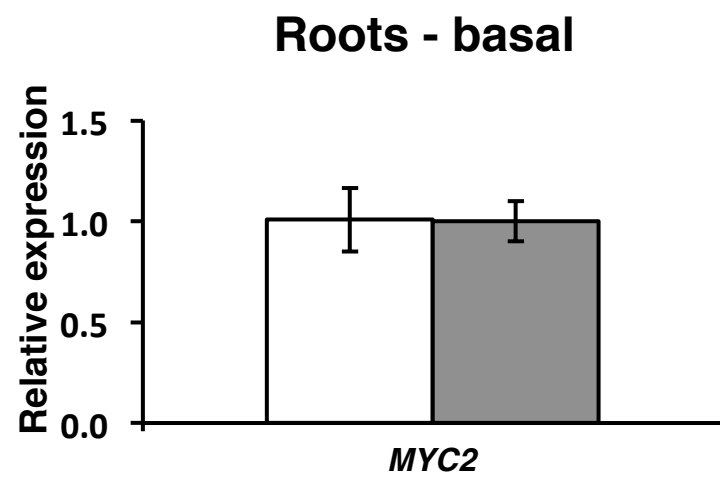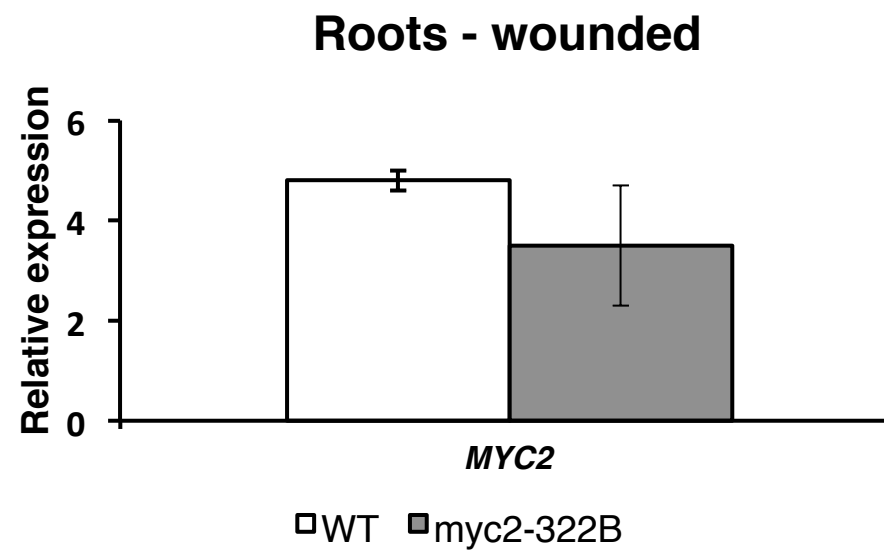

Supplement: S16 Fig — Transcript levels were normalized to those of UBC21 and displayed relative to the expression in the unwounded WT control. Bars represent the means of three biological replicates (±SD), each containing a pool of ~60 roots. (PDF) [file pgen.1005300.s016.pdf]

*MYC2<sub>pro</sub>-MYC2-CITRINE*

*MYC2<sub>pro</sub>-MYC2<sup>E165K</sup>-CITRINE*

**Mock**

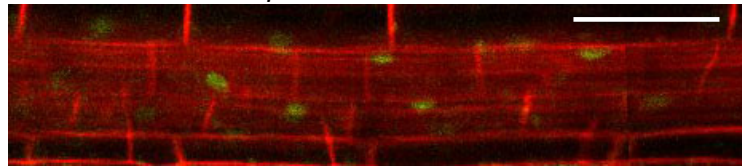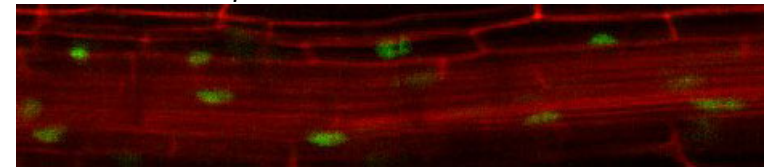

**CHX 30 min**

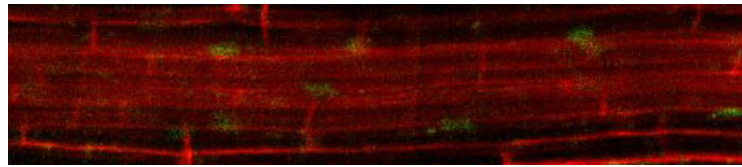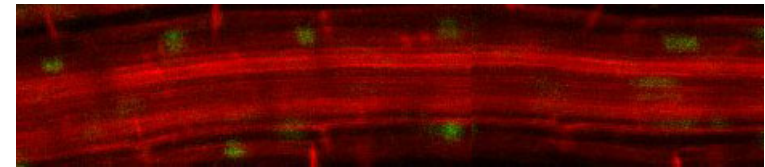

**CHX 60 min**

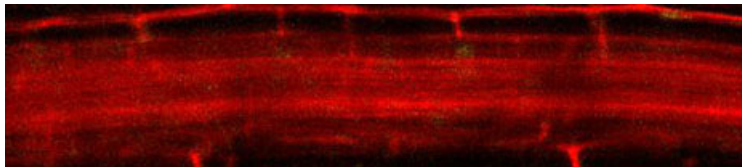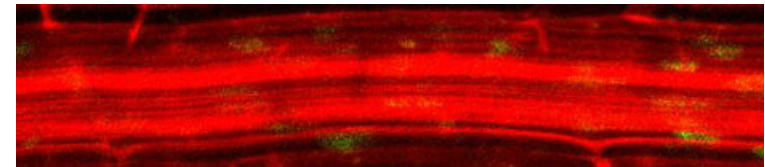

Supplement: S17 Fig — 5-do jin1-7 seedlings transformed with either MYC2 pro -MYC2-CITRINE or MYC2 pro -MYC2 E165K -CITRINE were treated with 100 μM CHX for the indicated times, after which primary roots were stained with propidium iodide (red) and examined by confocal microscopy. Details of the vascular tissues in the elongation zone where the florescent (green) signal was more intense are shown. The experiment was repeated three times with two independent lines for each reporter. Scale bar = 50 μm. Note: because the expression of chimeric proteins is under the control of MYC2 endogenous promoter, their expression level was too low to be detected in Western blots from 5-do seedlings. Moreover, although we analyzed 24 independent T2 lines, we failed to recover transgenic lines overexpressing MYC2E165K protein under the UBIQUITIN 10 promoter (At4g05320), suggesting that this protein version may be harmful to plants if expressed constitutively. (PDF) [file pgen.1005300.s017.pdf]

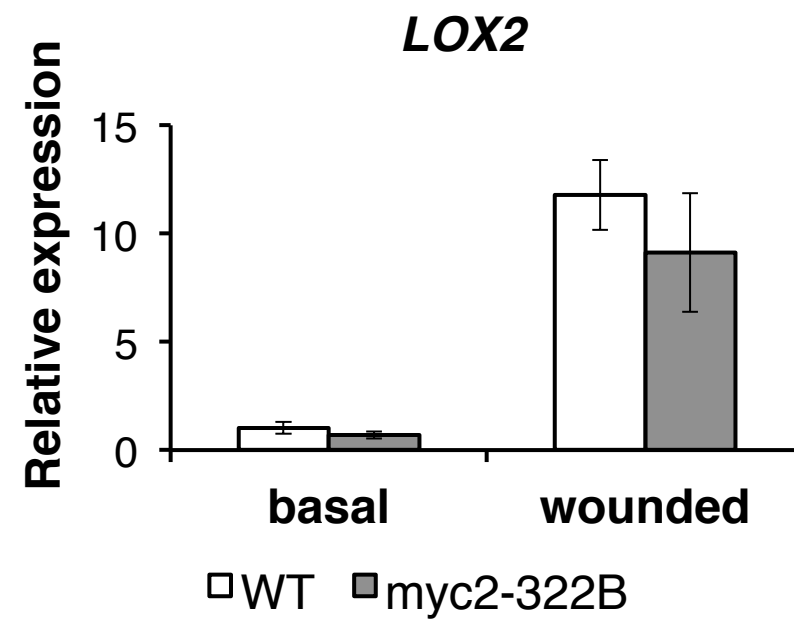

Supplement: S18 Fig — Transcript levels were normalized to those of UBC21 and displayed relative to the expression in the unwounded WT control. Bars represent the means of three biological replicates (±SD), each containing a pool of ~40 seedlings. (PDF) [file pgen.1005300.s018.pdf]

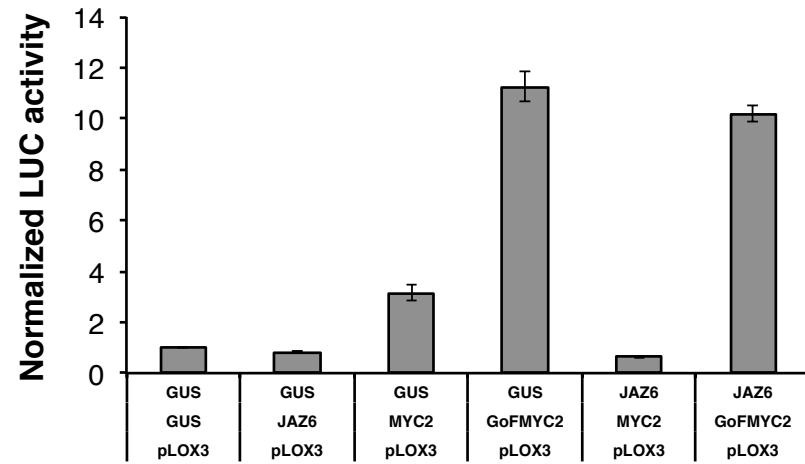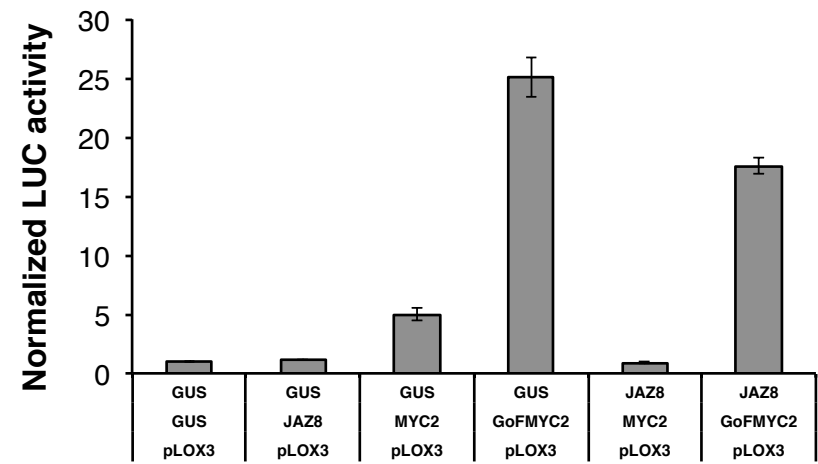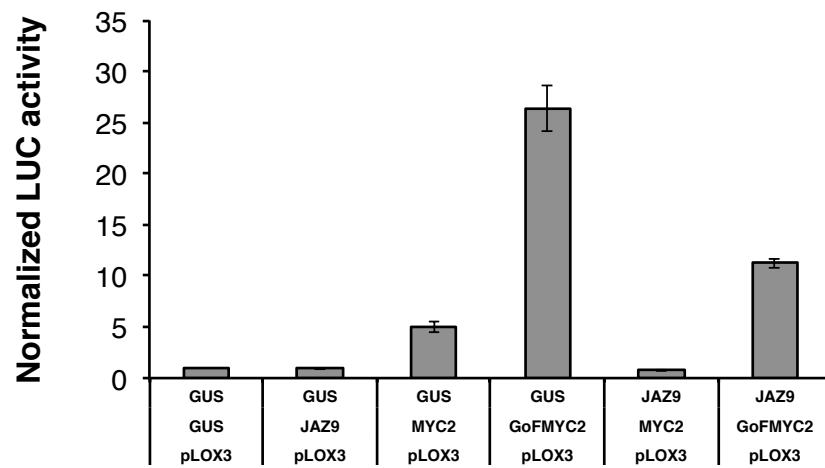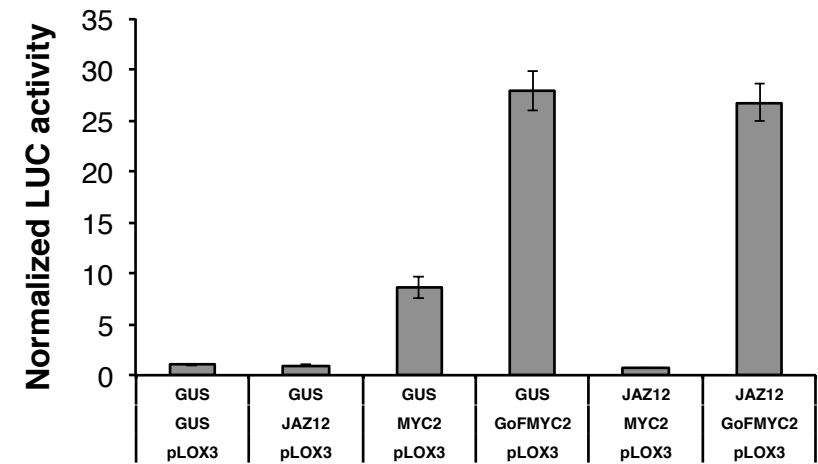

Supplement: S19 Fig — Tobacco protoplasts were transfected with a LOX3 pro -fLUC (LOX3p) reporter construct, a 35S pro -MYC2 (M2) or 35S pro -MYC2 E165K (M2E165K) effector constructs in the presence or absence of a 35S pro -JAZ6 (JAZ6), 35S pro -JAZ8 (JAZ8), 35S pro -JAZ9 (JAZ9) or 35S pro -JAZ12 (JAZ12) construct, and a 35S pro -rLUC normalization construct. The 35S pro -GUS (GUS) was used as control. Bars represent the means of 8 biological replicates (±SE) of normalized fLUC:rLUC activities. (PDF) [file pgen.1005300.s019.pdf]

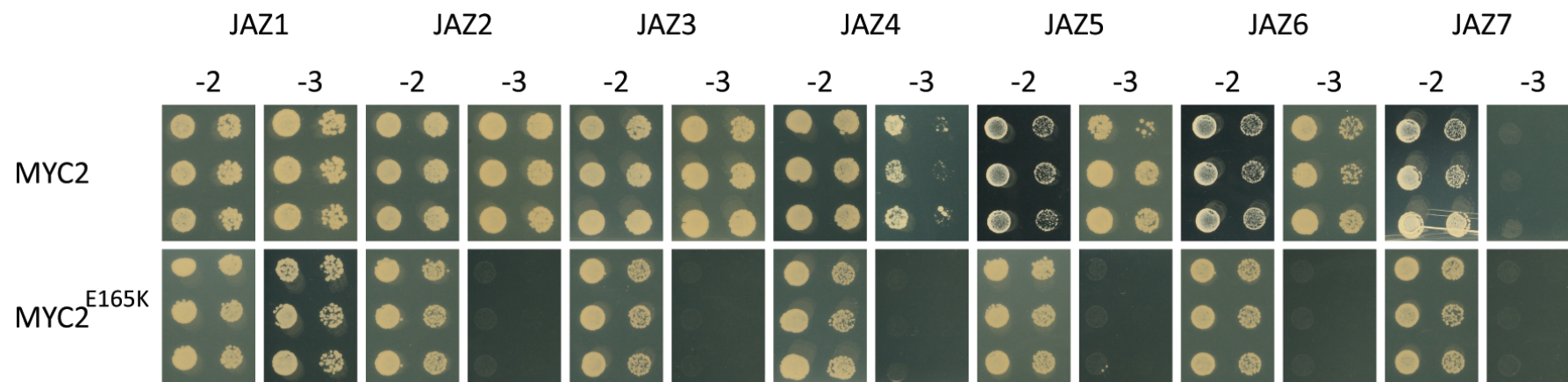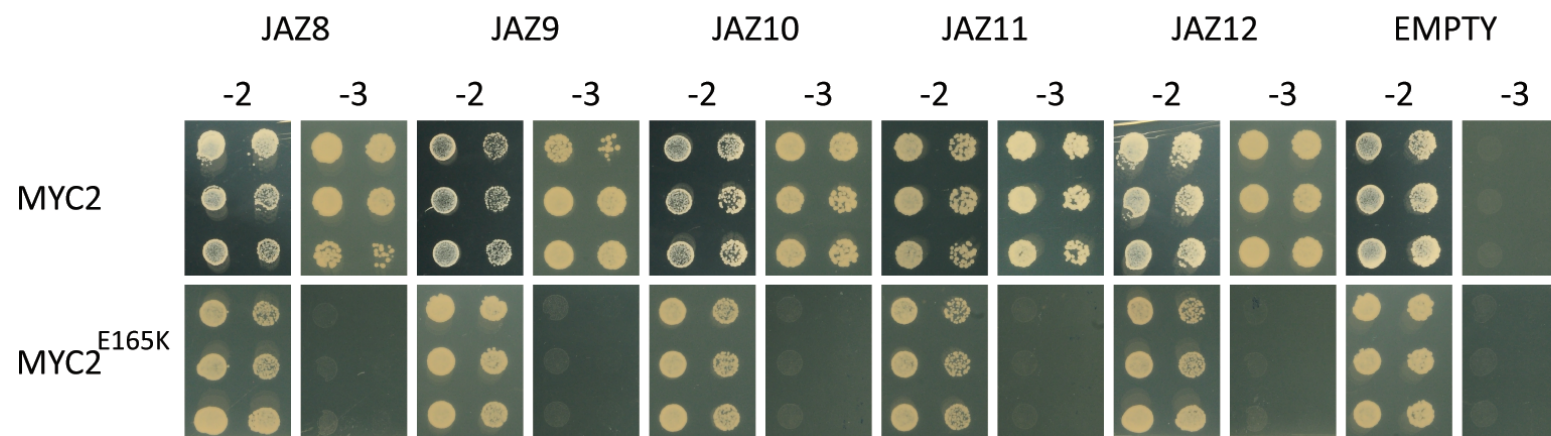

Supplement: S20 Fig — Yeast cells co-transformed with prey (MYC2 or MYC2E165K) and baits (JAZ1-12) were selected and grown on synthetic defined media lacking Leu and Trp (-2) as transformation control and on selective media lacking Leu, Trp and His (-3) to test protein interactions. (PDF) [file pgen.1005300.s020.pdf]

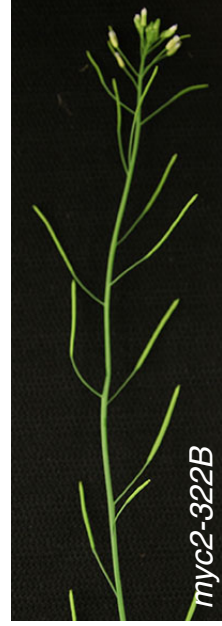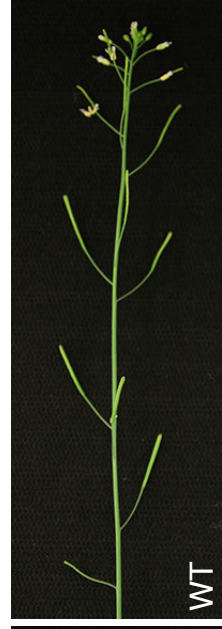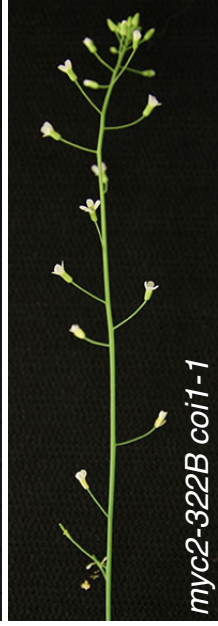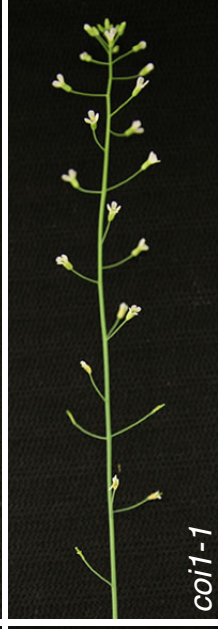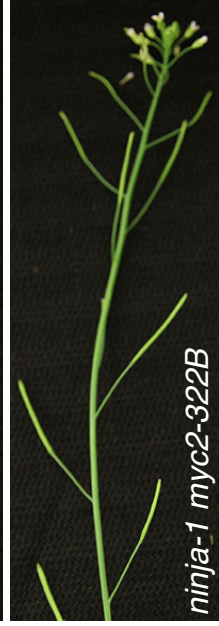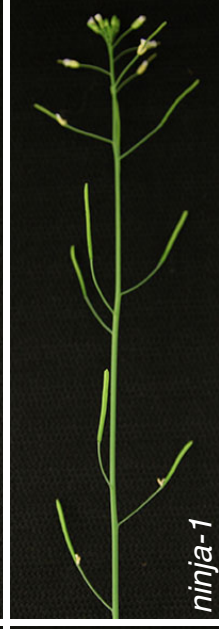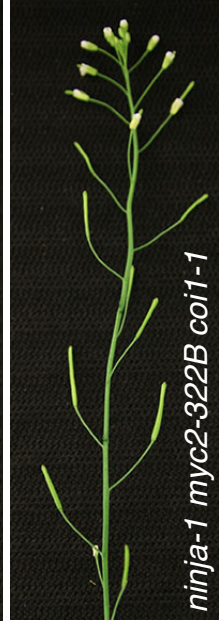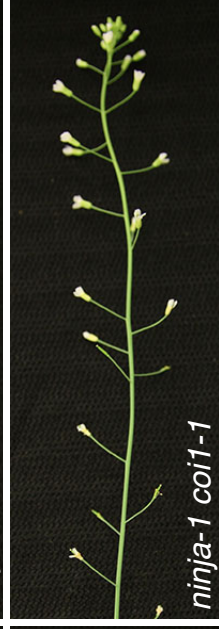

Supplement: S21 Fig — Main inflorescences from 5 week-old plants of indicated genotypes. Note the lack of sterility in the ninja-1 myc2-322B coi1-1 triple mutant. (PDF) [file pgen.1005300.s021.pdf]

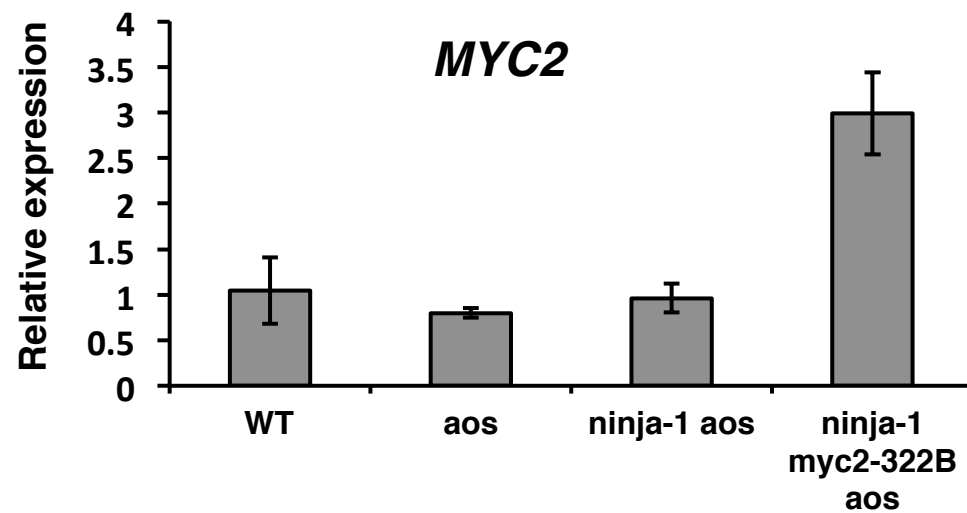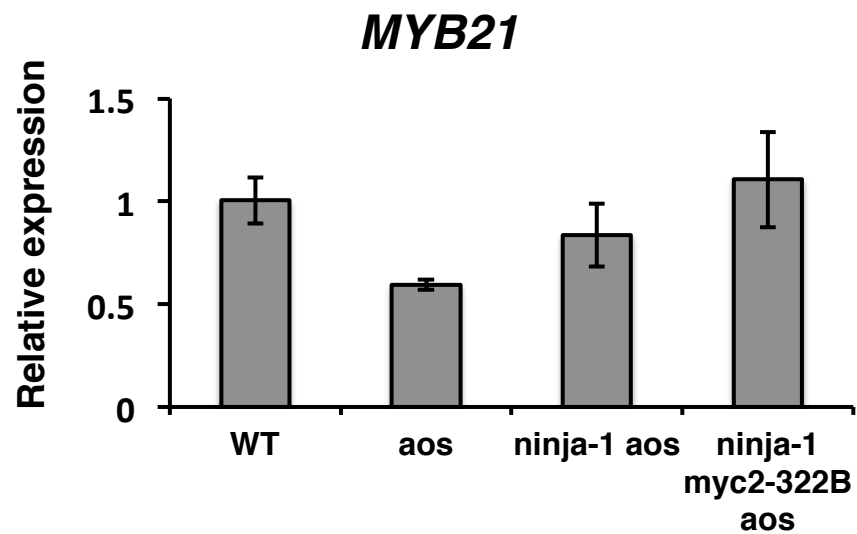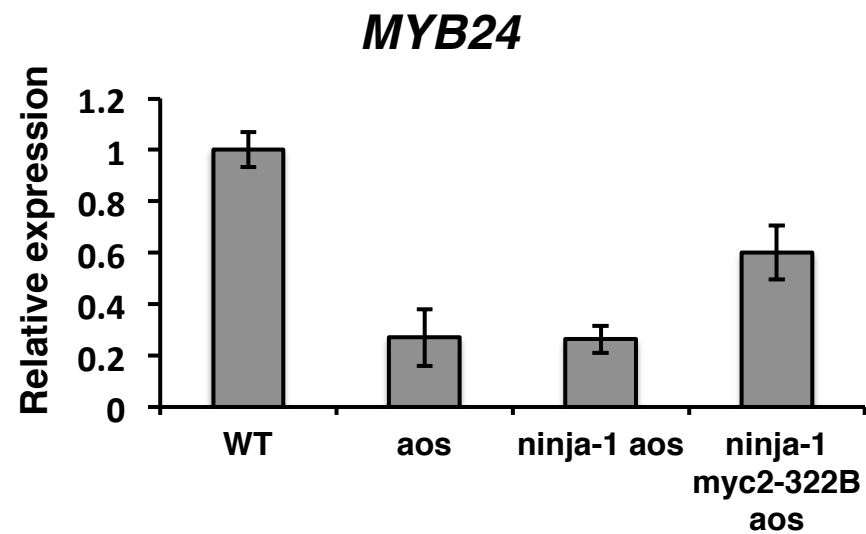

Supplement: S22 Fig — Transcript levels were normalized to those of UBC21 and displayed relative to the expression in the WT controls. Bars represent the means of three biological replicates (±SD), each consisting of equivalent stage 12 flower buds from 3–4 inflorescences of the same plant. (PDF) [file pgen.1005300.s022.pdf]

WT

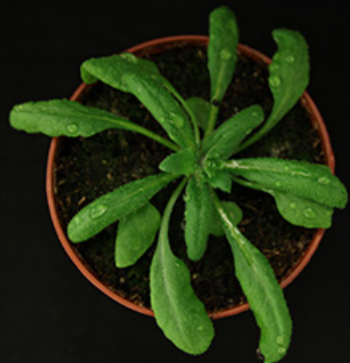

*aos*

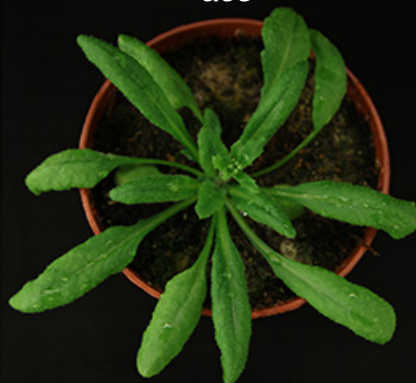

*ninja-1*

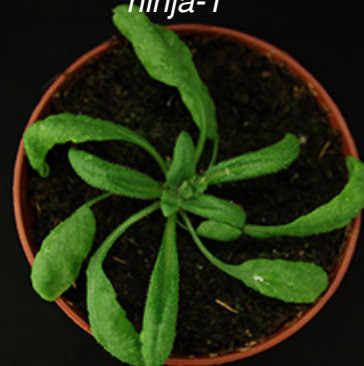

*ninja-1 aos*

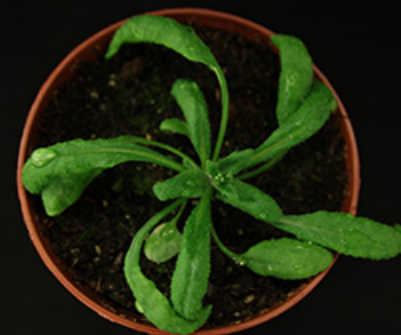

*myc2-322B*

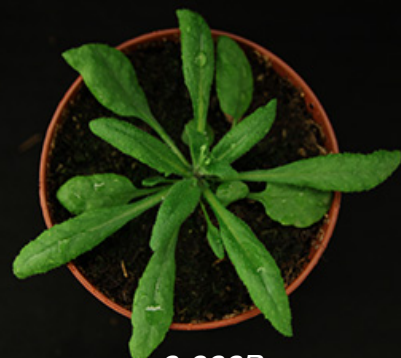

*myc2-322B aos*

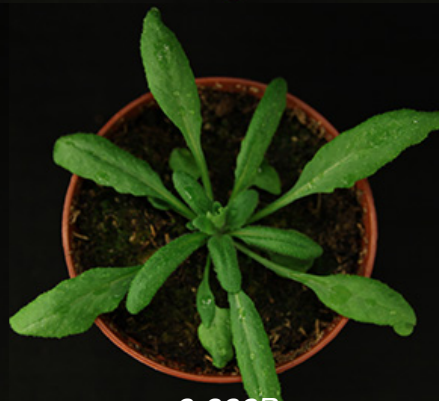

*ninja-1 myc2-322B*

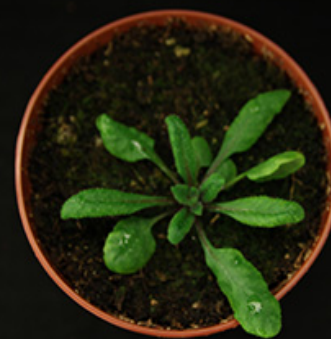

*ninja-1 myc2-322B aos*

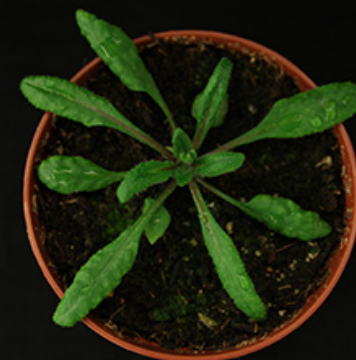

Supplement: S23 Fig — Plants were grown in continuous days for 4 weeks. Scale (diameter of each pot) = 7cm. (PDF) [file pgen.1005300.s023.pdf]

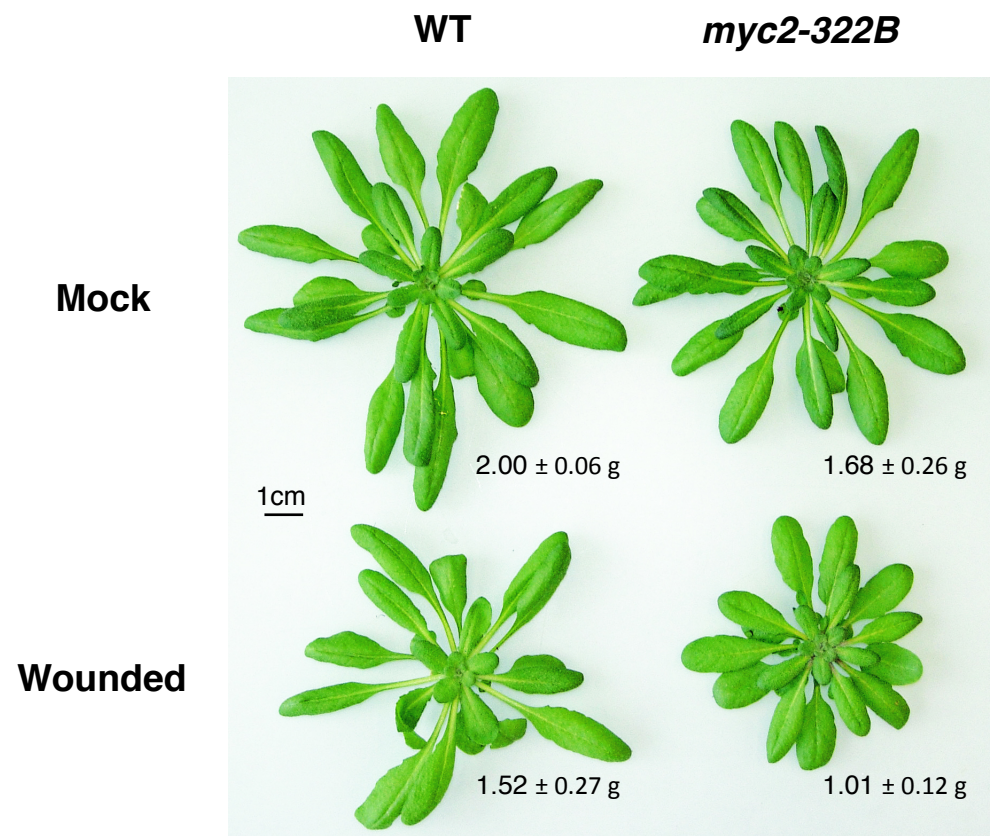

Supplement: S24 Fig — Rosette phenotypes of WT and myc2-322B plants grown in short days for 5 weeks. At the age of two weeks, plants were wounded 5 times on different leaves at 3-d intervals or gently touched on the same leaf (Mock). Leaves were treated in the following order: leaf 2 (L2), L4, L5, L6 and L8. Numbers below plants indicate rosette mean fresh weight ± SD, n = 6. (PDF) [file pgen.1005300.s024.pdf]

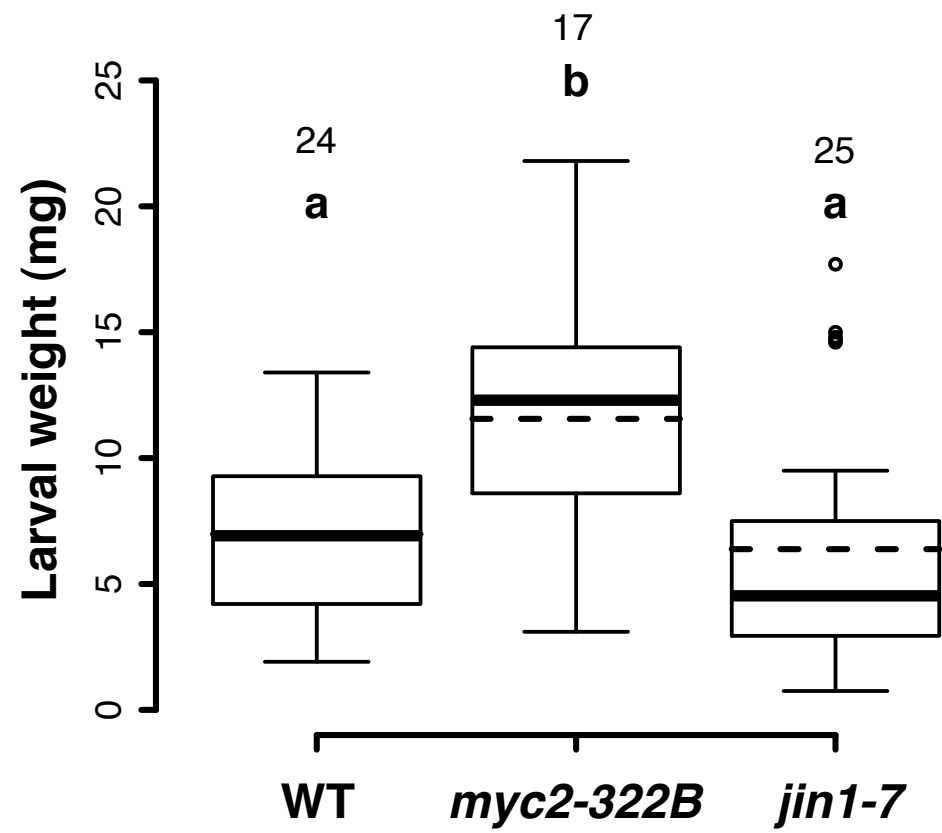

Supplement: S25 Fig — Medians and means are represented inside the boxes by solid and dotted lines respectively. Circles depict outlier data points beyond ±1.5X the interquartile range defined by the whiskers; numbers indicate n. Letters indicate statistically significant differences between pairs as determined by Tukey’s HSD test. (PDF) [file pgen.1005300.s025.pdf]
