# Supplementary material for: Multilayered Organization of Jasmonate Signalling in the Regulation of Root Growth
Source: PLoS Genet. 2015 Jun 12;11(6):e1005300. doi: 10.1371/journal.pgen.1005300 (PMC4466561; doi:10.1371/journal.pgen.1005300)
Supplement: S6 Fig — Images represent merged overlays of the fluorescent (yellow) and propidium iodide (red) stained roots. Scale bar = 50 m. (PDF) [file pgen.1005300.s006.pdf]

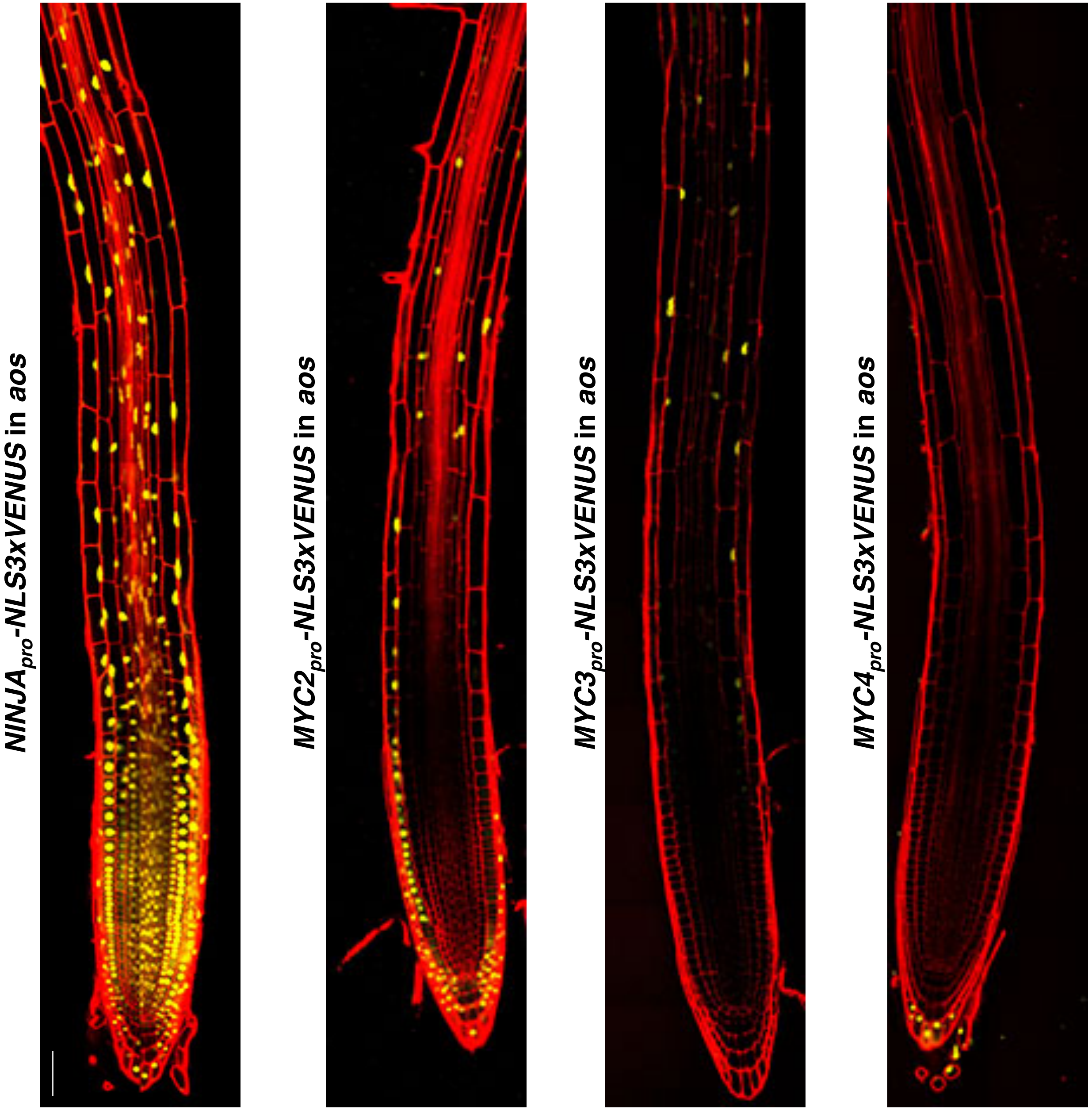

NINJA$_{pro}$-*NLS3xVENUS* in *aos*
MYC2$_{pro}$-*NLS3xVENUS* in *aos*
MYC3$_{pro}$-*NLS3xVENUS* in *aos*
MYC4$_{pro}$-*NLS3xVENUS* in *aos*
