# Supplementary material for: Multilayered Organization of Jasmonate Signalling in the Regulation of Root Growth
Source: PLoS Genet. 2015 Jun 12;11(6):e1005300. doi: 10.1371/journal.pgen.1005300 (PMC4466561; doi:10.1371/journal.pgen.1005300)
Supplement: S1 Table — (DOCX) [file pgen.1005300.s026.docx]

| **S1 Table.** Differentially expressed genes between 5-do *ninja-1* and WT roots | | | |
| --- | --- | --- | --- |
| **AGI code^A^** | **Fold Change^B^** | **p-value^C^** | **Description** |
| **JA signaling^D^** | | | |
| AT5G13220* | 33.4 | 0.0014 | JAZ10 |
| AT2G23620 | 8.9 | 0.0113 | methyl esterase 1 (MES1) |
| AT1G44350 | 5.4 | 0.0084 | IAA-leucine resistant (ILR)-like gene 6 (ILL6) |
| AT1G72450 | 4.8 | 0.0134 | JAZ6 |
| AT1G17380 | 4.1 | 0.0451 | JAZ5 |
| AT1G74950 | 3.3 | 0.0075 | JAZ2 |
| AT1G19180 | 2.9 | 0.0044 | JAZ1 |
| AT5G07010 | 2.4 | 0.0398 | sulfotransferase 2A (ST2A) |
| AT2G06050 | 2.4 | 0.0084 | oxophytodienoate-reductase 3 (OPR3) |
| AT5G42650 | 2.2 | 0.0438 | allene oxide synthase (AOS) |
| AT1G78660 | 2.1 | 0.0334 | gamma-glutamyl hydrolase 1 (GGH1) |
| AT4G28910* | -5.5 | 0.0044 | NINJA |
| **Transcription factors** | | | |
| AT5G22570 | 7.2 | 0.0114 | WRKY38 |
| AT4G37850 | 4.3 | 0.0479 | basic helix-loop-helix bHLH025 |
| AT5G61430 | -3.6 | 0.0300 | NAC domain containing protein 100 (NAC100) |
| **Secondary metabolism and cell wall** | | | |
| AT5G48000 | 58.5 | 0.0037 | thalianol hydroxylase 1, CYP708A (THAH1) |
| AT5G24140 | 28.1 | 0.0007 | squalene monooxygenase 2 (SQP2) |
| AT5G48010 | 25.3 | 0.0018 | thalianol synthase 1 (THAS1) |
| AT5G42580 | 21.9 | 0.0036 | cytochrome P450 705A12 |
| AT1G66800 | 18.3 | 0.0115 | alcohol dehydrogenase |
| AT5G38020 | 16.4 | 0.0050 | carboxyl methyltransferase |
| AT5G47990 | 9.9 | 0.0044 | thalian-diol desaturase 1, CYP705A5 (THAD1) |
| AT1G21100 | 7.2 | 0.0015 | indole glucosinolate O-methyltransferase 1 (IGMT1) |
| AT5G42590 | 5.4 | 0.0114 | marneral oxidase, CYP71A16 (MRO) |
| AT3G51450* | 3.8 | 0.0110 | Calcium-dependent phosphotriesterase superfamily protein |
| AT5G47980 | 3.7 | 0.0112 | HXXXD-type acyl-transferase family protein |
| AT3G46700 | 3.1 | 0.0071 | UDP-Glycosyltransferase superfamily protein |
| AT3G16390 | 3.1 | 0.0132 | nitrile specifier protein 3 (NSP3) |
| AT5G47950 | 2.9 | 0.0076 | HXXXD-type acyl-transferase family protein |
| AT4G24340 | 2.8 | 0.0037 | Phosphorylase superfamily protein |
| AT5G23220 | 2.7 | 0.0441 | nicotinamidase 3 (NIC3) |
| AT3G32030 | 2.5 | 0.0451 | Terpenoid cyclases/Protein prenyltransferases superfamily protein |
| AT2G41480 | 2.5 | 0.0274 | Peroxidase superfamily protein |
| AT1G26250 | 2.4 | 0.0451 | Proline-rich extensin-like family protein |
| AT4G38770 | 2.0 | 0.0112 | proline-rich protein 4 (PRP4) |
| AT3G47010 | 2.0 | 0.0454 | Glycosyl hydrolase family protein |
| **Oxidation - reduction processes** | | | |
| AT3G59710 | 21.6 | 0.0063 | NAD(P)-binding Rossmann-fold superfamily protein |
| AT1G03410 | 12.8 | 0.0016 | oxygenase superfamily protein (2A6) |
| AT1G14120 | 10.1 | 0.0110 | oxygenase superfamily protein |
| AT3G55290 | 8.3 | 0.0084 | short-chain dehydrogenase/reductase isoform D (SDRD) |
| AT5G36220 | 5.4 | 0.0044 | cytochrome P450 81D1 |
| AT1G06620* | 4.7 | 0.0084 | 2-oxoglutarate (2OG) Fe(II)-dependent oxygenase superfamily protein |
| AT5G05600* | 4.4 | 0.0064 | 2-oxoglutarate (2OG) Fe(II)-dependent oxygenase superfamily protein |
| AT3G03190 | 4.1 | 0.0496 | glutathione S-transferase F11 (GSTF11) |
| AT1G28480 | 3.8 | 0.0381 | Thioredoxin superfamily protein (GRX480) |
| AT4G21830 | 3.0 | 0.0259 | methionine sulfoxide reductase B7 (MSRB7) |
| AT1G69930 | 3.0 | 0.0084 | glutathione S-transferase TAU 11 (GSTU11) |
| AT4G23420 | 2.8 | 0.0081 | NAD(P)-binding Rossmann-fold superfamily protein |
| AT3G29250 | 2.8 | 0.0091 | NAD(P)-binding Rossmann-fold superfamily protein (SDR4) |
| AT1G50560 | 2.7 | 0.0071 | cytochrome P450 705A25 |
| AT5G63450 | 2.6 | 0.0481 | cytochrome P450 94B1 |
| AT4G04840 | 2.6 | 0.0016 | methionine sulfoxide reductase B6 (MSRB6) |
| AT5G20550 | 2.3 | 0.0101 | 2-oxoglutarate (2OG), Fe(II)-dependent oxygenase superfamily protein |
| AT1G64900 | 2.3 | 0.0181 | cytochrome P450 89A2 |
| AT4G15330 | 2.3 | 0.0191 | cytochrome P450 795A1 |
| At3g20940 | 2.2 | 0.0275 | cytochrome P450 705A31P |
| AT3G09940 | 2.0 | 0.0214 | monodehydroascorbate reductase (MDHAR) |
| AT1G14540 | -4.1 | 0.0084 | peroxidase 4 (PER4) |
| AT1G26390 | -4.7 | 0.0236 | FAD-binding Berberine family protein |
| **Stress responses** | | | |
| AT2G43530 | 9.6 | 0.0051 | defensin-like family protein |
| AT2G39310 | 6.5 | 0.0036 | jacalin-related lectin 22 (JAL22) |
| AT2G38750 | 4.6 | 0.0016 | annexin 4 |
| AT1G14960 | 4.3 | 0.0385 | Polyketide cyclase/dehydrase and lipid transport superfamily protein |
| AT2G34930 | 4.0 | 0.0103 | disease resistance family protein/ LRR family protein |
| AT3G59730 | 3.6 | 0.0193 | L-type lectin receptor kinase V.6, LECRK-V.6 |
| AT2G38760 | 3.3 | 0.0398 | annexin 3 |
| AT2G43535 | 3.3 | 0.0114 | defensin-like (DEFL) family protein |
| AT5G35940 | 2.9 | 0.0240 | Mannose-binding lectin superfamily protein |
| AT2G26740 | 2.6 | 0.0454 | soluble epoxide hydrolase (SEH) |
| AT1G71695 | 2.3 | 0.0467 | Peroxidase superfamily protein |
| AT1G05200 | 2.3 | 0.0123 | glutamate receptor 3.4 (GLR3.4) |
| AT4G22212 | 2.3 | 0.0097 | defensin-like protein |
| AT3G16450 | 2.1 | 0.0085 | Jacalin-related lectin 33 (JAL33) |
| AT1G55020 | 2.0 | 0.0114 | lipoxygenase 1 (LOX1) |
| AT2G01520 | -2.3 | 0.0259 | MLP-like protein 328 (MLP328) |
| AT5G06760 | -2.6 | 0.0025 | Late Embryogenesis Abundant 4-5 (LEA4-5) |
| AT1G78820 | -2.9 | 0.0473 | D-mannose binding lectin protein |
| AT2G35980 | -7.5 | 0.0275 | Yellow-leaf-specific gene 9 (YLS9) |
| **Transport** | | | |
| AT5G02170 | 7.6 | 0.0114 | Transmembrane amino acid transporter family protein |
| AT4G21903 | 3.5 | 0.0070 | MATE efflux family protein |
| AT5G40210 | 3.5 | 0.0132 | nodulin MtN21 /EamA-like transporter family protein (UMAMIT42) |
| AT5G38030 | 3.5 | 0.0050 | MATE efflux family protein |
| AT1G16370 | 3.2 | 0.0114 | organic cation/carnitine transporter 6 |
| AT5G65990 | 3.0 | 0.0275 | Transmembrane amino acid transporter family protein |
| AT1G22550 | 2.8 | 0.0047 | Major facilitator superfamily protein |
| AT3G45650 | 2.8 | 0.0084 | nitrate excretion transporter1 (NAXT1) |
| AT3G16690 | 2.3 | 0.0273 | SWEET16 |
| AT4G27860* | 2.1 | 0.0189 | Membrane of ER body 1 (MEB1) |
| AT3G47960 | 2.0 | 0.0159 | glucosinolate transporter-1 (GTR1) |
| AT3G45680 | -3.0 | 0.0275 | Major facilitator superfamily protein |
| **Lipid metabolism** | | | |
| AT4G22610 | 33.8 | 0.0058 | Bifunctional inhibitor/ lipid-transfer protein |
| AT3G02610 | 17.6 | 0.0041 | stearoyl-acyl-carrier-protein desaturase family protein |
| AT5G23840 | 12.7 | 0.0095 | MD-2-related lipid recognition domain-containing protein |
| AT4G12550 | 4.1 | 0.0112 | Auxin-Induced in Root cultures 1 (AIR1) |
| AT4G33110 | 3.7 | 0.0259 | methyltransferases superfamily protein |
| AT1G70810 | 2.8 | 0.0080 | Calcium-dependent lipid-binding (CaLB domain) family protein |
| **Protein degradation** | | | |
| AT1G35625 | 11.5 | 0.0025 | RING/ U-box superfamily protein |
| AT1G79310 | 6.2 | 0.0064 | metacaspase 7 (MC7) |
| AT5G19110 | 6.1 | 0.0085 | aspartyl protease family protein |
| AT5G38710 | 4.8 | 0.0479 | Methylenetetrahydrofolate reductase family protein |
| AT1G35330 | 3.2 | 0.0014 | RING/ U-box superfamily protein |
| AT1G73310 | 2.2 | 0.0309 | serine carboxypeptidase-like 4 (SCPL4) |
| AT4G09110 | -4.2 | 0.0133 | RING/U-box superfamily protein |
| AT4G11310 | -4.2 | 0.0050 | Papain family cysteine protease |
| **Other metabolism** | | | |
| AT3G06020 | 3.1 | 0.0250 | FANTASTIC FOUR 4 (FAF4) |
| AT4G29700 | 2.9 | 0.0084 | Alkaline-phosphatase-like family protein |
| AT1G24320 | 2.8 | 0.0177 | Six-hairpin glycosidases superfamily protein |
| **Other hormonal responses** | | | |
| AT3G02875 | 2.6 | 0.0250 | IAA-Leu resistant 1 |
| AT4G39030 | 2.3 | 0.0037 | Enhanced disease susceptibility 5 (EDS5) |
| AT2G24400 | -2.0 | 0.0348 | SAUR-like auxin-responsive protein family |
| **Unclassified or unknown** | | | |
| AT2G18210 | 5.0 | 0.0064 | unknown protein |
| At2G17330 | 4.6 | 0.0114 | pseudogene |
| At4g13860 | 4.4 | 0.0181 | RNA-binding (RRM/RBD/RNP motifs) family protein |
| AT3G59340 | 4.1 | 0.0079 | Eukaryotic protein of unknown function (DUF914) |
| AT3G07350 | 3.8 | 0.0228 | Protein of unknown function (DUF506) |
| AT5G26270* | 3.5 | 0.0084 | unknown protein |
| AT5G23830 | 3.1 | 0.0076 | MD-2-related lipid recognition domain-containing protein |
| AT5G23820 | 2.5 | 0.0275 | MD-2-related lipid recognition domain-containing protein |
| AT2G26530 | 2.5 | 0.0454 | Protein of unknown function (DUF1645) |
| AT4G16146 | 2.4 | 0.0110 | cAMP-regulated phosphoprotein 19-related protein |
| AT4G29690 | 2.2 | 0.0132 | Alkaline-phosphatase-like family protein |
| AT2G46150 | 2.1 | 0.0077 | Late embryogenesis abundant (LEA) glycoprotein family |
| AT5G26260 | 2.1 | 0.0417 | TRAF-like family protein |
| AT5G27930 | 2.1 | 0.0228 | Protein phosphatase 2C family protein |
| ^A^ differentially expressed genes between untreated 5-do roots of WT and the *ninja-1* mutant organized by gene onthology (GO) functional classes. In some cases genes can fall into more than one category. Genes with an asterisk were in common with the *NINJA* RNAi adult rosette transcriptome (Pauwels et al., 2010).  ^B^ Fold Change of the means of three biological replicated experiments. A negative number indicates down regulated genes.  ^C^ False Discovery rate (FDR) corrected p-value.  ^D^ Other transcripts typically involved in JA responses (*LOX6*, *MYC2*, and *JAZ3*) were also significantly up-regulated, but their expression levels were below the chosen cut-off value of >2 fold change (within a range of 1.98 and 1.84 fold change). | | | |
